# Modularity of Online Social Networks and COVID-19 Misinformation Spreading in Russia: Combining Social Network Analysis and National Representative Survey

**DOI:** 10.2196/58302

**Published:** 2025-06-26

**Authors:** Boris Pavlenko

**Affiliations:** 1 International Center for the Study of Institutions and Development HSE University Moscow Russian Federation

**Keywords:** COVID-19, Russia, infodemic, modularity, online social networks, VK, misinformation, beliefs, information spreading, social network analysis

## Abstract

**Background:**

The outbreak of SARS-CoV-2 in 2019 was accompanied by a rise in the popularity of conspiracy theories. These theories often undermined vaccination efforts. There is evidence that the spread of misinformation about COVID-19 is associated with online social media use. Online social media enables network effects that influence the dissemination of information. It is important to distinguish between the effects of using social media and the network effects that occur within the platform.

**Objective:**

This study aims to investigate the association between the modularity of online social networks and the spread of, as well as attitudes toward, information and misinformation about COVID-19.

**Methods:**

This study used data from the social network structure of the online social media platform Vkontakte (VK) to construct an adjusted modularity index (fragmentation index) for 166 Russian towns. VK is a widely used Russian social media platform. The study combined town-level network indices with data from the poll “Research on COVID-19 in Russia’s Regions” (RoCIRR), which included responses from 23,000 individuals. The study measured respondents’ knowledge of both fake and true statements about COVID-19, as well as their attitudes toward these statements.

**Results:**

A positive association was observed between town-level fragmentation and individuals’ knowledge of fake statements, and a negative association with knowledge of true statements. There is a strong negative association between fragmentation and the average attitude toward true statements (*P*<.001), while the association with attitudes toward fake statements is positive but statistically insignificant (*P*=.55). Additionally, a strong association was found between network fragmentation and ideological differences in attitudes toward true versus fake statements.

**Conclusions:**

While social media use plays an important role in the diffusion of health-related information, the structure of social networks can amplify these effects. Social network modularity plays a key role in the spread of information, with differing impacts on true and fake statements. These differences in information dissemination contribute to variations in attitudes toward true and fake statements about COVID-19. Ultimately, fragmentation was associated with individual-level polarization on medical topics. Future research should further explore the interaction between social media use and underlying network effects.

## Introduction

### Background

The COVID-19 pandemic challenged many societies. Governments had to reduce mortality risks while minimizing economic shocks. One of the most efficient ways to mitigate such impacts is vaccination. There is considerable variation in people’s behavior regarding vaccination [1]. Evidence suggests that one of the factors associated with increased vaccine hesitancy was the use of social networking and belief in COVID-19 fake news and conspiracy theories [2-4].

Pivetti et al [5] proposed a mechanism that links social media usage, COVID-19 fake news, and vaccination hesitancy. First, online social media users are more likely to encounter misinformation about COVID-19 compared with traditional media users [6]. Second, exposure to fake news increases the likelihood that some users will believe in conspiracy theories [3]. Belief in conspiracy theories, in turn, raises the perceived risk of vaccination while lowering the perceived risk of the disease [7]. Finally, these altered risk perceptions reduce the likelihood of vaccination and negatively impact other preventive behaviors [8,9].

However, a causal relationship between online social media use and belief in conspiracy theories has not yet been established. Moreover, there is evidence that the unavailability of social media positively influences the popularity of searches for COVID-19 fake news [10]. Finally, social media can also promote proscience or provaccine messages that reduce vaccination hesitancy [11,12]. In other words, what matters is the content observed on social media, not social media use itself.

It is important to study the Russian case of COVID-19 pandemic attitudes for several reasons. Russia exhibited relatively high levels of vaccine hesitancy [1], which may have contributed to one of the highest excess mortality rates in the world [13,14]. Additionally, the availability of data and variation at both the individual and town levels offer valuable tools for examining the issue.

Theories concerning the consumption of information on online social media generate mixed hypotheses. On the one hand, prior beliefs may be reinforced by filter bubbles created by social networking site algorithms that tailor users’ feeds to match their interests [15]. Echo chambers further enable individuals to interact primarily with others who share similar views [16]. On the other hand, individuals may also encounter differing perspectives online [17], which can increase the diversity of their views.

The spread of misinformation about COVID-19 is supported by echo chamber theory and evidence that homogeneous groups are more likely to disseminate fake news [18-20]. However, when information escapes an echo chamber, only a minority of outsiders accept it [18]. According to the opinion dynamics model [21], individuals are likely to change their beliefs only when the ideological gap with the person they interact with is small. Similarly, there are homogeneous groups that disseminate scientific information as well [16]. Previous review studies have shown that it is primarily human users, rather than bot accounts, who spread fake news to most users [22], underscoring the importance of studying the networks of ordinary users.

This paper provides evidence on the indirect effects of online social media on misinformation diffusion and the formation of personal beliefs. The study findings indicate that it is not only the platform that matters but also the structure of citizens’ online interpersonal networks. In this study, data on the characteristics of online social media networks on Vkontakte (VK) were combined with polling data from users in the corresponding towns. It was shown that town-level fragmentation of these networks is associated with the spread of misinformation about COVID-19 and the share of fake statements encountered by respondents. The results remain robust after controlling for individual characteristics, including the use of online and traditional media, fear of COVID-19, and household experience with the disease. This study contributes to the growing literature on the spread of health misinformation on social media [23].

The presented approach differs from studies that rely solely on online social media data. Numerous studies have examined the spread of specific conspiracy theories within online environments [23]. However, a key question remains: how does the social environment—measured through a network index—affect an individual’s likelihood of encountering true or false statements about COVID-19? Finally, how do networks shape opinions about these statements, and what role does online social media play in this process?

### Related Work

This section is divided into a brief literature review covering studies on information diffusion in social networks, network modularity, and its outcomes. These areas are explored to understand how information spreads within networks and how modularity influences social outcomes. Following this, the estimation of fragmentation will be discussed and the formulation of hypotheses will be presented based on the literature review and methodological approach.

### Information Spread in the Network

Information diffusion refers to the process of spreading information among agents or communities within a network [24]. The efficiency of this diffusion is influenced by the network’s structure and the characteristics of its nodes and links. The concept of the “strength of ties” introduces the role of interpersonal trust between individuals in a network [25]. Strong ties exist between nodes that share many neighbors, while weak ties connect nodes with fewer common neighbors. In social interactions, strong ties typically form between individuals who know each other well. While strong ties are more effective for persuading people within the network, weak ties can facilitate broader and more efficient information spread [26]. Homophily—the tendency of similar nodes to group together based on shared traits—also enhances the speed of information diffusion within these clusters [27]. At the structural level, the concept of communities is central; communities are groups of nodes clustered based on their connectivity [28]. Modularity, which measures the strength of the division of a network into communities, plays a significant role in shaping how information diffuses [29-31]. The foundational theoretical framework for studying information diffusion is the Epidemic Spread Model [32].

All of these theories are interdependent; for example, higher homophily is expected among nodes within the same community. In other words, individuals with more ties to each other are likely to share common characteristics. In this study, no personal information about the nodes in the network was used, which limits the range of analytical tools available. Instead, the modularity index was used to measure potential polarization within a town [29].

### Network’s Modularity and Its Outcomes

Network modularity influences the speed of information flow within a network [30]. In animal networks, modularity has been shown to affect the efficiency of information transmission in a nonlinear manner [31]. Similarly, in the context of COVID-19, the structure of the network influences disease spread even when the total number of links remains constant [33]. Strategies that incorporate knowledge of community structure significantly improve the efficiency of containment efforts at the town level [33-35].

Previous studies have examined the fragmentation of online social networks and the spread of misinformation within them. For a review of the spread of fake news and belief change, see [36]. These studies have shown that individuals within the same community are more likely to influence each other’s opinions than outsiders. A concept particularly relevant to this study is that of epistemic echo chambers [37], which arise when individuals rely primarily on their social networks for information, and when those networks are fragmented—thereby limiting exposure to diverse viewpoints.

Several studies have examined the relationship between network modularity and economic or social outcomes. In this context, modularity is used as a tool to capture social capital. For instance, the modularity of networks at the municipal level has been shown to predict economic development [38], while at the town level, it has been linked to levels of corruption [39]. More broadly, network modularity serves as one indicator for predicting societal polarization at the network level [29].

Most studies have focused on how misinformation about COVID-19 spreads online, how social media use influences vaccination intentions [3], or knowledge of specific conspiracy theories [18]. For a review of studies on this topic, see [3,40].

What distinguishes this study from previous work is its focus on the indirect effects of network modularity on the town-level spread of information and misinformation. Specifically, the author posits that the likelihood of epistemic echo chambers emerging in a town is correlated with the fragmentation of its network. The study estimates the association between the fragmentation of VK’s social media network at the town level and the likelihood of individuals encountering fake news and related opinions, while controlling for individual characteristics. The study findings suggest that online social media polarization and fragmentation can have broader consequences, extending beyond individual opinion formation to affect information dynamics at the community level.

## Methods

### Fragmentation Index as Network’s Modularity Measure

As shown previously, network structure plays a key role in the transmission of information. At the macro level, the focus shifts to communities, where understanding how members are interconnected becomes essential. To capture this, a modularity index was used as a measure of network interconnectedness. In this study, an improved version of the modularity index was applied to better account for differences in network size.

The *fragmentation index* indicates the extent to which nodes tend to cluster together and remain separate from others. The Louvain algorithm [41] divides a network into relatively segregated groups (communities). It should be noted that the problem of community detection is NP-hard; therefore, the algorithm provides a heuristic solution. The fragmentation index estimates the likelihood that edges occur within small groups rather than between them.



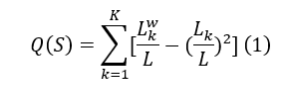



Equation 1 represents the modularity index of the network that captures the ratio of ties with and between community members, where *L* is the number of edges in the network, *L_k_* is the number of edges adjacent to members of community *k*, *L_k_^w^* is the number of edges within community *k*, and *Q*(*S*) is the modularity of the network.



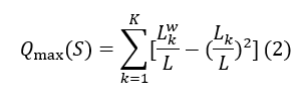



Equation 2 shows the adjustment for modularity calculated on the alternative network if all ties were within community members, where *Q*_max_(*S*) is the theoretical maximum if all edges were within the communities.

Equation 3 represents the fragmentation index:

*F*_s_=[*Q*(*S*)]/[*Q*_max_(*S*)] (3)

In other words, the fragmentation index is a modularity measure adjusted for network size, allowing for meaningful comparison of network structures across different sizes. This methodology follows previous research in the field [39,42].

The modularity index has been shown to influence the spread of information [30,31,43]. Moreover, it has been established that a network’s modular structure, as measured by the modularity index, affects the spread of diseases [33]. Both processes are modeled using the same basic theoretical framework [32].

### Hypothesis Formulation

Higher fragmentation indicates that there are more connections within communities than between them. In such networks, information is more likely to circulate within a community rather than spread across communities. If we assume that community members share certain characteristics that make them more similar to one another than to outsiders, they are more likely to agree with the information they encounter and to disseminate it further [18]. This, in turn, increases the likelihood of repeated exposure to the same information.

Building on the theory of the strength of weak ties [25], the diversity-bandwidth trade-off—comparing the properties of weak ties with strong ties—can also be applied at the community level [26]. Existing literature shows that both conspiracy theories and scientific news tend to spread within closed, homogeneous groups [16,44]. In more fragmented networks, such groups are more likely to exist, as higher homogeneity increases the probability that individuals belong to the same community. While both weak and strong ties can facilitate the spread of new information, the effectiveness depends on the type of information and the surrounding environment [26]. In the context of online news dissemination, the prevalence of strong ties does not necessarily offer an advantage—unless trusted users (those with higher bandwidth) are more likely to believe in and share accurate information.

- Hypothesis 1(a): Fragmentation is negatively associated with the relative number of true statements about COVID-19 encountered.

Lower fragmentation implies the presence of more weak ties, which can facilitate the spread of accurate information in the context of news dissemination. In less fragmented networks, individuals who believe in fake news are more likely to be connected to others with differing views, increasing the chances of exposure to corrective information.

In other words, while less fragmented networks tend to exhibit a greater diversity of views, the likelihood of encountering fake information online is lower. This is because individuals who believe in fake news are less likely to share it in such environments, as the audience response to these posts tends to be weaker. This behavior can be explained by the concept of strategic self-presentation [45,46]. Users are more likely to share content that portrays them favorably and garners positive feedback from others. In diverse networks, posts expressing extreme views are likely to be received less favorably due to greater ideological distance between the poster and the audience. As a result, more neutral content is amplified, as it attracts broader approval. While this mechanism is particularly pronounced on online platforms due to algorithmic reinforcement [36], it also holds relevance in offline social interactions.

- Hypothesis 1(b): Fragmentation is positively associated with the relative number of fake statements about COVID-19 encountered.

In towns with more fragmented networks, individuals are more likely to be divided into numerous closed communities. This structure fosters epistemic echo chambers, where exposure to diverse viewpoints is limited [37]. Within such communities, the spread of fake news and conspiracy theories is more likely to occur, increasing the chances that individuals connected to or adjacent to these communities will encounter misinformation.

- Hypothesis 2(a): Fragmentation is negatively associated with belief in true statements about COVID-19.

Groups within fragmented networks are more likely to share similar views and exert strong influence on one another’s opinions [36]. That is, individuals within the same community tend to hold similar beliefs. However, different communities within the same network may hold opposing views, contributing to overall polarization. Within individual communities, lower diversity of opinion, fewer weak ties, and reduced efficiency of information flow may limit exposure to accurate information. As a result, individuals in more fragmented networks may be less informed and less likely to believe true statements.

- Hypothesis 2(b): Fragmentation is positively associated with belief in fake statements about COVID-19.

In more fragmented networks, the spread of fake statements is more likely, as the existence of closed communities where misinformation is accepted and reinforced becomes more probable. Individuals within or adjacent to these communities are more likely to encounter and believe such statements, which are then further propagated. However, the overall effect may be limited. When fake news circulates beyond these echo chambers, it often encounters resistance or negative reactions. Such exposure can prompt critical evaluation among those outside the community, leading to skepticism and potential revisions in belief [18].

### Causality

The methodology used in this study does not permit causal inference between network fragmentation, the likelihood of encountering information, and an individual’s belief in that information. However, reviews of prior research offer insights into potential causal mechanisms linking information spread and opinion change [36]. Previous studies indicate that fragmented networks are prone to increasing polarization [47], and the dissemination of polarized content can further intensify opinion divergence [48]. Additionally, exposure to opposing viewpoints may reinforce existing beliefs—a phenomenon known as belief entrenchment [49]—whereas exposure to more neutral information can reduce polarization and shift opinions [21].

Summing up, in fragmented networks, the emergence of closed groups that believe in fake news is more likely due to the formation of epistemic echo chambers. Strong believers within these groups may share misinformation beyond their immediate community. However, such exposure is unlikely to change the views of others—particularly if those individuals already hold conflicting beliefs.

### Data

The dataset combines the “Research on COVID-19 in Russia’s Regions” (RoCIRR) database on COVID-19 in Russia with social network data collected from the most popular online social network, VK. The RoCIRR dataset includes responses from over 23,000 individuals. Data were collected between November 4 and December 1, 2020, through an online survey of respondents from 61 Russian regions, designed to be representative at the regional level. Detailed information about the poll is provided in Multimedia Appendix 1. Respondent recruitment was conducted by Online Market Intelligence, an online polling company operating in Russia and analogous to Amazon’s Mechanical Turk (MTurk) in the United States. Online Market Intelligence maintains a panel of the adult population in cities with over 100,000 residents. In addition, a subsample of respondents was drawn from smaller towns and rural areas for the RoCIRR database. For more details on the RoCIRR dataset, see [50].

From this dataset, towns with more than 10 respondents were selected, resulting in 168 towns. For these towns, VK users were identified using VK API instruments [51] to construct social networks. Additional data sources included official municipal statistics from Rosstat [52] and the main regional socioeconomic statistics from Rosstat [53], which were used to create town-level controls for wages and population. Municipal statistics were missing for 2 towns due to changes in municipality structure; detailed information about this is provided in Multimedia Appendix 2. All responses marked as “difficult to say” were treated as missing observations and excluded from the survey. The final dataset included 16,587 respondents from 166 towns, with an average of 119.2 respondents per town and 88,700 nodes per network. The dataset covered 60 of the 61 regions included in the original sample.

The final dataset includes both smaller towns with populations under 100,000 and major cities such as Moscow and Saint Petersburg. Most towns in the sample (95/166) have populations below 250,000, though large cities (with populations over 500,000) are also represented. The sample comprises both regional capitals and ordinary towns. A detailed distribution of town populations in the sample is provided in Multimedia Appendix 3.

### About VK

VK is a Russian online social network where users can post public messages, add other users to their “friend” lists, and send private messages. As of November 2023, VK was the fifth most popular website in Russia [54] and the most popular online social network, with over 80 million monthly active users.

Data from VK were collected using the VK API between February 7 and February 25, 2023. It is assumed that networks based on VK data remain relatively stable over time. To support this assumption, 2 samples of the fragmentation index from 2023 and 2024 were used and statistical differences were assessed. It should be noted that community detection algorithms are heuristic, and therefore different runs may result in different node groupings [55]. As a result, observed differences in fragmentation could arise solely from the algorithm’s allocation process. Descriptive statistics for the datasets are provided in Multimedia Appendix 4. A paired *t* test (1-tailed) shows no significant differences between the fragmentation indexes from 2023 and 2024 (*P*=.07). Moreover, it has been shown that the use of retrospective data in online social network analysis is possible, though its effectiveness may be limited by account bans [56].

This section explains the algorithm used to select accounts from VK. For each town, the algorithm attempts to identify accounts based on the following criteria: age group (3 age groups ranging from 18 to 65 years), gender, and number of friends (at least 100 from the same town and no more than 500 in total). In total, accounts are selected from 6 groups—3 age groups, each split by gender. The algorithm was run 3 times, resulting in the selection of up to 18 accounts per town. It should be noted that in larger towns, it is easier to find accounts that meet these criteria due to the larger pool of available accounts. Thus, this method allows for the construction of networks with sizes roughly proportional to the actual population sizes of the towns. The criteria were chosen to simplify calculations, filter out bot accounts at an early stage, and prioritize accounts belonging to individuals likely residing in the towns they list. The threshold of 100 friends is consistent with the mode number of friends observed on platforms such as Facebook and Twitter [57]. Statistical characteristics are presented in Table 1.

**Table 1:** Characteristics of networks (n=166).

Statistic	Mean (SD)	Range
Fragmentation index	0.449 (0.093)	0.293-0.799
Number of nodes	88,749.800 (81,418.540)	375-541,123
Number of edges	688,859.500 (487,270.800)	2783-2,463,411
Density	0.001 (0.003)	0.00001-0.040
Clustering	0.225 (0.075)	0.030-0.442

**Figure 1:**
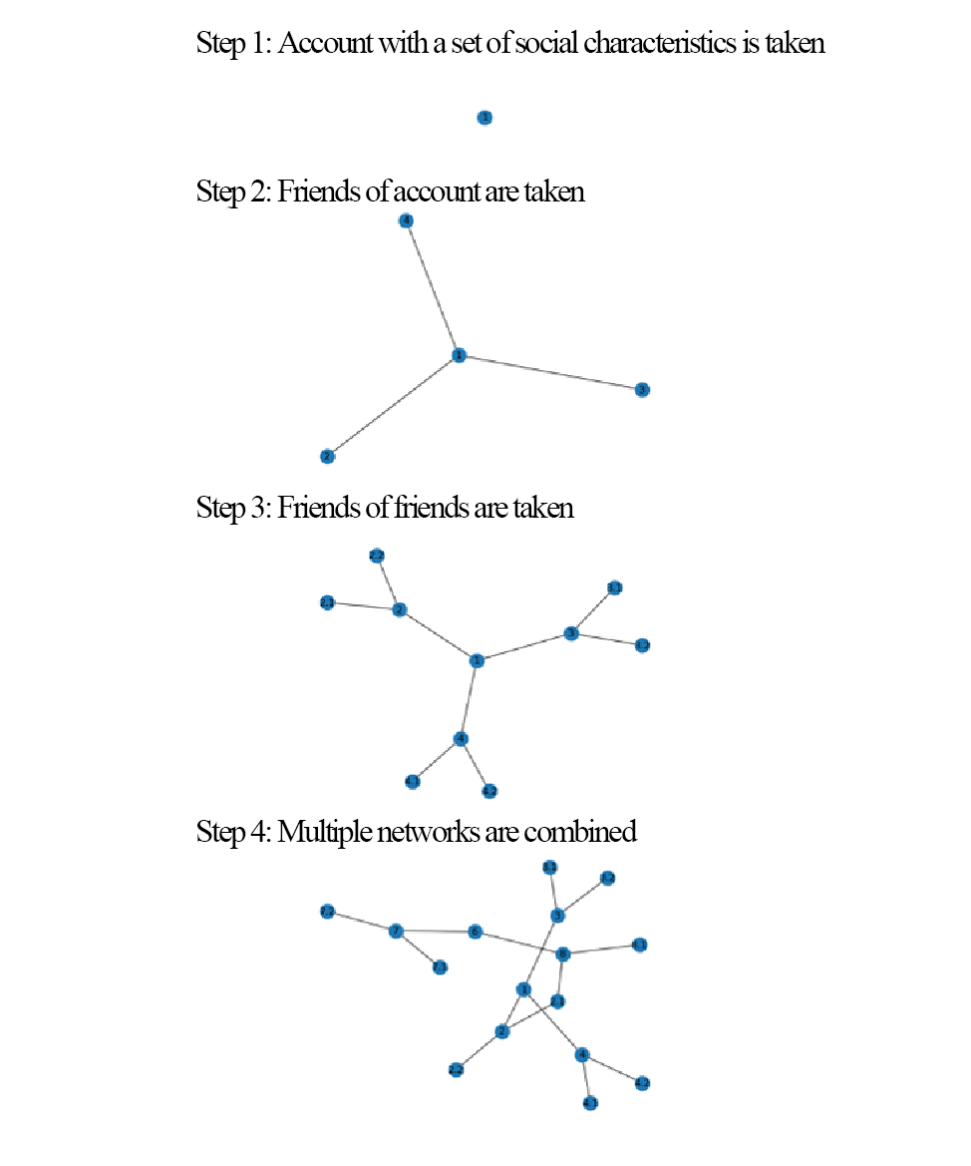
Network data collection procedure.

Networks for each account are sampled using breadth-first search to a depth of 2. In other words, for each selected account, their friends and the friends of those friends are included—but acquaintances of friends of friends are not sampled. A network representation of the data collection procedure is shown in Figure 1. While breadth-first search introduces a sampling bias toward high-degree nodes, this bias decreases as the proportion of sampled nodes increases [58]. Furthermore, by using multiple accounts to construct each network, randomness is introduced into the sampling process, which helps reduce potential bias [59].

To estimate node and edge coverage, the author proposes calculating proportions relative to the population as the lower limit of coverage, and proportions relative to VK users as the upper limit. The lower limit reflects the representation of a hypothetical social network encompassing all citizens, while the upper limit captures the representation of the actual VK user network. Based on this, the lower bound indicates that 88,750 of the 435,663 (20.37%) 2019 town populations were screened, suggesting that a significant portion of the population is represented in the network. The upper bound of node coverage—adjusted using the share of respondents who reported using VK—is estimated to be slightly over 30% (88,750/272,766, 32.54%). The lower limit of edge coverage is nearly 3% (688,859/21,783,150, 3.16%), representing the proportion of edges in the sampled graph relative to all social connections of citizens, with the Dunbar number used as the average number of edges [57]. The upper limit of edge coverage reflects the proportion of edges in the sampled network relative to the expected number of edges among VK users from the same town.

The upper limit of edge coverage was estimated as the product of the proportion of VK users in the total population and the average share of friends from the same town. These calculations yield an upper estimate of edge coverage of nearly 13% (688,859/5,416,402, 12.72%). Details on the estimation of the expected degree are provided in Multimedia Appendix 5. Previous studies have shown that sampling 15% of a graph is sufficient [60]. While the study’s sample is smaller, it is important to note that the effects of sampling bias on community structure are more limited than on other network characteristics. This is because high-degree nodes—which are more likely to appear in a sample than in the full network—are particularly relevant for determining network structure [61]. Therefore, reducing bias by increasing the share of low-degree nodes in a sample may actually harm the preservation of community structure [61]. Furthermore, the relationship between changes in the fragmentation index and the number of accounts sampled was analyzed, as detailed in Multimedia Appendix 5. As expected, an overlap among the combined networks was observed, resulting in diminishing returns in both edge coverage and node coverage as the network size increases. The most significant spikes in edge coverage occur when the first 3 networks are combined. On average, only a modest increase in edge coverage was observed after combining 10 networks. These estimations suggest that some bias may be introduced by the chosen sampling technique, although the study aims to demonstrate that this bias is minimal. Multimedia Appendix 5 presents edge coverage for each account selected in the sampling process. In a subsample of 49 towns, the average edge coverage reaches nearly 16% (543,687/3,406,978, 15.96%) after selecting 14 accounts and increases to over 20% (726,626/3,389,593, 21.44%) with 18 accounts. Note that the number of iterations to create each of the 49 networks varies by city. Thus, as we examine changes in the average edge coverage introduced by each iteration of sampling, the total number of towns in the sample may vary. However, even with all 18 accounts, some towns still show edge coverage below 10% (637,297/6,600,559, 9.66%). Findings from this study indicate that 15 of the 22 (68%) towns in the subsample achieve edge coverage above 24% (779,875/3,136,689, 24.86%) using this method. This suggests that some bias may result from the network sampling approach. To address this, a robustness check was conducted, restricting the analysis to towns with average edge coverage above 15% (739,677/4,917,172, 15.04%). The results are presented in Multimedia Appendix 6.

Note that it is possible for a person to have multiple accounts or to incorrectly state their hometown, and the poll sample may overrepresent social media users. Data on the number of network nodes for each town and corresponding population census figures are presented in Multimedia Appendix 3.

### Main Variables

The main variables of interest are the proportion of known fake and true statements about COVID-19, and the sum of attitudes toward fake and true statements. Additionally, a measure of polarization was constructed.

The control variables are social media usage (VK) and network size. Individual-level controls include gender (dummy), education level, household income, age, fear of COVID-19, a dummy variable indicating household experience with COVID-19, and institutional trust measured as trust in the president. The analysis also controls for television use, as these variables have been used in previous studies on attitudes toward COVID-19 beliefs [6,50]. At the town level, the analysis controls for the natural logarithm of average wage (2019) and the natural logarithm of population (2019). Descriptive statistics for all variables are provided in Table 2.

This study combines individual-level survey data from towns with the network characteristics of those towns. It is assumed that individuals from the same town are influenced by the characteristics of that town’s network, as constructed from VK data. A weaker assumption is that differences in VK users’ network structures reflect underlying mechanisms of network formation, such as social capital [62]. The estimates are therefore conservative—the study assumes that all respondents are equally affected by the level of fragmentation in their town. In the descriptive statistics (Table 2), town-level characteristics are weighted by the number of respondents in each town; such variables are marked accordingly.

**Table 2:** Descriptive statistics for used variables (n=16,587).

Statistic	Mean (SD)	Range
Fragmentation index (town level)	0.487 (0.103)	0.293 to 0.799
Gender dummy (1 is female)	0.620 (0.485)	0 to 1
Education level	4.247 (1.055)	1 to 5
Household income group	3.403 (0.820)	1 to 5
Age (years)	36.569 (9.664)	18 to 80
Network size (natural logarithm of the number of nodes; town level)	11.731 (0.790)	5.927 to 13.201
Number of nodes in the network (town level)	155,199.500 (93,322.270)	375 to 541,123
VK use dummy	0.617 (0.486)	0 to 1
Natural logarithm of real wages 2019 (town level)	10.743 (0.147)	10.191 to 11.262
Natural logarithm of population 2019 (town level)	13.310 (0.819)	10.497 to 16.350
Household COVID-19 experience dummy	0.124 (0.330)	0 to 1
Fear of COVID-19	2.936 (0.891)	1 to 4
Trust in president	3.020 (1.466)	1 to 5
Television as a source of news	0.566 (0.496)	0 to 1
Share of encountered fake statements about COVID-19	0.458 (0.286)	0.000 to 1.000
Share of encountered true statements about COVID-19	0.534 (0.257)	0.000 to 1.000
Attitude to true statements about COVID-19	2.143 (2.058)	–8 to 8
Attitude to fake statements about COVID-19	–2.223 (2.792)	–10 to 10
Share of true statements agreed	0.415 (0.257)	0.000 to 1.000
Share of fake statements agreed	0.105 (0.161)	0.000 to 1.000
Difference in attitude between fake and true statements	–4.366 (3.634)	–18 to 12
Misinformation error	0.184 (0.220)	0.000 to 1.750

This section of the paper provides a detailed overview of the primary questions included in the questionnaire used in this study. Table 3 presents the exact translated wording of these key questions. In the first question, which concerns the encounter with true and false statements, respondents are given a list of statements and asked to indicate which ones they have come across, with the option to select “none of the above.” In the following question, respondents are asked to indicate their attitudes toward the plausibility of the statements they had previously selected.

**Table 3:** Translated wordings of the main questions.

Question number	Question wording
Question 1: knowledge of statements	Select statements that you have previously come across
Question 2: attitude to statements (only for questions that the respondent chooses in the list in previous questions)	Rate your attitude to the following statement (Likert scale from 1 to 4, and difficult to answer): 1=The statement is absolutely NOT reliable; 4=The statement is absolutely reliable.

A measure of the spread of COVID-19–related information and misinformation was created. To do this, the number of encountered statements about COVID-19 was summed and the proportion relative to the total number of statements included in the questionnaire was calculated. These calculations are done separately for true and fake statements. As a result, the proportion of encountered true statements and the proportion of encountered fake statements were obtained. Calculating proportions allows us to consider the full set of statements rather than analyzing each one individually. The relationship between the network’s fragmentation at the town level and the individual-level encounter with information was interpreted as an indicator of the average level of information spread within a city.

To examine whether information exposure translates into differences in beliefs, attitudes toward the statements were calculated. The relevant survey question measures the extent to which respondents believe a given statement is true. Attitudes were quantified by transforming responses on the Likert scale (originally ranging from 1 to 4) to a scale from –2 to +2, but only for the statements the respondent recognized. If a respondent had never encountered a statement or found it difficult to answer, the corresponding value was set to 0. Then, the transformed scores for all true and fake statements were summed separately to form the true attitude and fake attitude variables. These transformations were designed to create an opinion variable that neutralizes agreement and disagreement across statements, while retaining observations even when a respondent skipped or was unsure about a particular item. Descriptive statistics for attitudes toward each statement are provided in Table 4. Statements 1-5 are classified as fake, and statements 6-9 as true.

**Table 4:** Attitude toward COVID-19 statements (n=16,587).

Statistic	Mean (SD)	Range	Never came across, n
1. COVID-19 does not exist	–1.219 (1.158)	–2 to 2	570
2. COVID-19 was developed by the United States	–0.112 (0.807)	–2 to 2	10,998
3. COVID-19 was developed by China	–0.229 (0.910)	–2 to 2	8775
4. 5G towers affect COVID-19 immunity	–0.448 (0.906)	–2 to 2	11,083
5. COVID-19 development was financed by Bill Gates	–0.214 (0.728)	–2 to 2	12,911
6. Russia has already developed its first vaccine	0.860 (1.096)	–2 to 2	3651
7. The Prime Minister of Russia got sick with COVID-19	0.570 (0.963)	–2 to 2	8497
8. Russian Government manipulates official COVID-19 statistics	0.674 (1.024)	–2 to 2	6161
9. Trump takes hydroxychloroquine to prevent getting COVID-19	0.040 (0.393)	–2 to 2	15,220

The share of statements a respondent agrees with was also calculated. This is done by summing binary variables coded as 1 if the respondent agrees (either strongly or weakly) and 0 if they do not. The total is then divided by the number of true or fake statements, respectively. This variable does not differentiate between strong and weak agreement, as that distinction is already captured by the attitude variable. Instead, it is used to examine whether fragmentation is associated with a higher overall share of agreed-upon statements, regardless of the strength of the respondent’s stance.

Finally, to estimate the overall levels of misinformation about COVID-19 associated with fragmentation, 2 additional variables were created. First, a measure of the difference between opinions on true and fake statements was constructed, which captures individual-level polarization in beliefs about COVID-19. Second, a total misinformation error score, defined as the sum of 2 components, was calculated: the share of fake statements a respondent agrees with and the share of true statements they disagree with.

VK use was measured using a binary variable indicating whether a respondent reported using the VK social media platform. Network size was calculated as the natural logarithm of the number of nodes in the combined network for each town. This variable serves as an alternative measure of VK’s relative popularity, derived from network data rather than self-reported survey responses or census statistics.

The final model that estimates the association with COVID outcomes is as follows:

COVID_outcomes_i_ = α + β_1_Fragmentation_j_ + β_2_VK_i_ + Controls_i_ + Controls_j_ + ε_i_

where *i* is the respondent from town *j*; COVID_outcomes*_i_* is the share of known statements, attitude measures, differences in attitude toward true and fake statements, and misinformation error; Fragmentation*_j_* is a town-level fragmentation measure; VK*_i_* is a dummy for VK online social media usage; Controls*_i_* is a set of individual controls; and Controls*_j_* is a set of town-level controls.

The model assumes that the estimated value of the fragmentation index is uniform across all respondents within a given town. The relationship between fragmentation and individual responses is treated as linear, allowing us to estimate the average effect of fragmentation at the town level. While this study does not differentiate the effects of network fragmentation between VK users and nonusers, it does estimate the direct effects of VK usage separately.

### Ethical Considerations

Only public information was collected from social media for this study. All relevant information about social networks is presented in an aggregated form and users cannot be reidentified. The survey used in the study was approved by the Columbia Institutional Review Board (protocol number IRB-AAAT4453).

## Results

### Overview

The results are presented in 2 sections: (1) the relationship between fragmentation and the spread of information, and (2) the relationship between fragmentation and attitudes toward statements. The first section demonstrates that network fragmentation is associated with differential patterns in the dissemination of true versus false statements. The second section highlights how fragmentation contributes to a widening gap between belief in misinformation and agreement with accurate statements about COVID-19, suggesting that fragmented online social networks play a role in shaping polarized beliefs.

The observed effect is primarily driven by a strong negative association between network fragmentation and attitudes toward true statements about COVID-19, while the coefficient for attitudes toward fake statements is statistically insignificant (*P*=.55).

### Part 1: Fragmentation and Information Spreading

Results in Table 5 indicate that the fragmentation index is positively associated with the number of encountered fake statements and negatively associated with the number of encountered true statements. Both findings support the initial hypotheses regarding the relationship between tie strength and information diffusion. Although the estimated coefficients are relatively small, they apply uniformly to all individuals within a given town. An increase in fragmentation from the lowest to the highest level observed in the sample would result in a 0.028 increase in the proportion of known fake statements—equivalent to approximately 64% (0.028/0.044) of the estimated effect of VK social media use. For true statements, the effect is even more pronounced, with a coefficient of –0.034, which exceeds the estimated effect of social media usage. Notably, the coefficients for VK social media use are both significant (*P*<.001) and positive, suggesting that online platforms facilitate the dissemination of information, regardless of its veracity.

**Table 5:** Information spreading.^a,b,c^

Information spread	Dependent variables		
	Share of encountered fake statements about COVID-19	Share of encountered true statements about COVID-19	
Fragmentation index	0.057 (0.028); .04	–0.068 (0.025); .006	
Size of the network	–0.016 (0.004); <.001	–0.004 (0.003); .18	
Vkontakte use dummy	0.044 (0.005); <.001	0.030 (0.004); <.001	
Socioeconomic town-level controls^d^	+	+	
Individual-level controls^e^	+	+	
Observations, n	16,587	16,587	
*R^2^*	0.018	0.037	

^a^Data for the first 3 rows are presented as estimated β coefficients of linear regression (SE); *P* value.

^b^Exact *P* values are reported.

^c^Robust SEs by town are given in brackets.

^d^Socioeconomic town-level controls include the natural log of wages 2019 and the natural log of population 2019.

^e^Individual-level controls include gender dummy, education level, household income, age, fear of COVID-19, a dummy variable for household experience with COVID-19, institutional trust measured as a trust to the president, and use of television.

### Part 2: Fragmentation and Attitude Toward Statements

Columns 1 and 2 of Table 6 show that fragmentation is associated with a lower average attitude toward true statements, while no significant relationship is observed for fake statements (*P*=.55). These serve as the baseline results for the association between network fragmentation and belief in true and fake COVID-19 statements. However, a more nuanced analysis is needed to understand the specific ways in which fragmentation influences belief. In particular, it is important to differentiate between respondents who moderately agree with several statements and those who strongly agree with only one. To capture this distinction, this study uses the share of statements with which a respondent agrees as an additional measure in the estimation.

If we look into columns 3 and 4, we observe that, for true statements, fragmentation leads to a lower share of people agreeing with true statements, but shows an insignificant relationship for fake statements (*P*=.12). Also, we observe that social media use is significantly negatively associated with attitude toward fake statements (*P*<.001), but at the same time, it is positively associated with the share of fake statements the respondent believes are true.

The difference in results between true and fake statements can be explained by the idea of ideological distance. When fake news spreads beyond its bubble of believers, it is eventually observed by those who hold different prior beliefs or have been exposed to different information. This leads to double-checking by people outside the initial bubble, which results in a stronger antifake stance. In other words, in more fragmented towns, fake news is spread more widely, but it does not translate into changes in beliefs. Similarly, VK users have a significantly lower average attitude toward fake news but agree with a higher share of fake statements. The result for VK use corresponds with the findings of Bursztyn et al [63], who show that VK use leads to greater variation in beliefs. While they attribute this to network echo-chamber effects, the relationship may be more complex, as no significant relationship was found between network fragmentation and the popularity of extreme opinions about COVID-19.

When looking at the combined results for differences in opinions about fake and true statements, polarization introduced by fragmentation was observed. Column 5 shows the difference between respondents’ views regarding fake and true statements. A change in fragmentation from the lowest level (Kostroma) in the sample to the highest (Astrakhan) is associated with a 0.5-point increase in the difference between attitudes. This corresponds to a nearly 14% (0.5/3.64) increase relative to the SD of the variable. At the same time, VK use decreases polarization, although the coefficient is smaller.

Finally, column 6 shows that fragmentation is positively associated with misinformation error—that is, agreeing with untrue statements and disagreeing with true statements. This indicates that, while the overall difference in attitude toward fake statements is indistinguishable from 0 (column 2), fragmentation leads to errors in respondents’ ability to distinguish between true and fake statements. VK use is also positively associated with misinformation errors, suggesting that people on online social media are less likely to distinguish fake news from real.

Summing up the results, the findings show that fragmentation is associated with the spread of misinformation and leads to disbelief in true statements, greater polarization of opinions, and increased misinformation errors.

**Table 6:** Relationship between the fragmentation of networks and attitudes toward statements.^a,b,c^

Statement relationship	Dependent variables					
	Average attitude to true statements about COVID-19	Average attitude to fake statements about COVID-19	Share of true statements agree	Share of fake statements agree	Difference in attitude between fake and true statements	Misinformation error
Fragmentation index	–0.848 (0.194); <.001	0.150 (0.274); .55	–0.102 (0.024); <.001	0.025 (0.016); .12	0.998 (0.355); .005	0.052 (0.022); .02
Size of the network	0.030 (0.027); .27	0.041 (0.038); .28	–0.0001 (0.003); .97	–0.003 (0.002); .13	0.011 (0.050); .82	–0.005 (0.003); .01
Vkontakte use dummy	0.055 (0.034); .10	–0.161 (0.046); <.001	0.022 (0.004); .001	0.006 (0.003); .04	–0.216 (0.060); <.001	0.014 (0.004); <.001
Socioeconomic town-level controls^d^	+	+	+	+	+	+
Individual-level controls^e^	+	+	+	+	+	+
Observations, n	16,587	16,587	16,587	16,587	16,587	16,587
*R* ^2^	0.077	0.058	0.059	0.025	0.069	0.027

^a^Data for the first 3 rows are presented as estimated β coefficients of linear regression (SE); *P* value.

^b^Exact *P* values are reported.

^c^Robust SEs by town are given in brackets.

^d^Socioeconomic town-level controls include the natural log of wages 2019 and the natural log of population 2019.

^e^Individual-level controls include gender dummy, education level, household income, age, fear of COVID-19, a dummy variable for household experience with COVID-19, institutional trust measured as a trust to the president, and use of television.

## Discussion

### Principal Findings

This study adds to the body of literature on the COVID-19 infodemic [40,64,65] and health misinformation more broadly [23]. The study’s findings show how the characteristics of the network in which an individual is embedded can lead to both polarization and the spread of misinformation. Moreover, network formation itself may be influenced by cultural and social traits of society, such as social capital [62]. This suggests that, more generally, some societies are structurally more prone to misinformation, and that online social media may amplify these effects by reinforcing polarization.

The study shows that social media users are more likely to be aware of both true and fake news, but the association is stronger for fake news. At the same time, social network fragmentation is positively associated with knowledge of fake news and negatively associated with knowledge of true statements. The combined impact of social media use and network fragmentation reveals a difference in the likelihood of encountering true versus fake news about COVID-19. For example, a respondent from the town with the highest fragmentation who uses VK is aware of 7.3% (0.0895-0.0167) more fake statements but 0.4% (–0.0243 to 0.0199) fewer true statements compared with a respondent who does not use social media and lives in the town with the lowest fragmentation. This result provides empirical evidence of differences in the spread of true and fake statements beyond online social media [28].

The study goes beyond medical research and contributes to the literature on the relationship between online social media and ideological polarization. Bursztyn et al [63] proposed a theoretical mechanism explaining how the distribution of preferences shifts with the penetration of social media. The study results align with the model’s predictions—echo chamber effects resulting from fragmentation increase ideological distance in opinions about true and fake statements. At the same time, social media use reduces this distance. Thus, the study offers a potential mechanism through which social media penetration leads to polarization. In fragmented networks, echo chambers are more prominent, which amplifies differences in opinions. As a result, the effects of online social network usage may be more constrained when accounting for the structure of such networks.

The study findings highlight the distinction between the effects of online social media use and those of social network structure. Previously, the effects of social media use and social media penetration were often interpreted as consequences of social media bubbles [63]. These are distinguishable correlations that can move in different directions. However, understanding the interrelation between social media use (or penetration) and network structure was beyond the scope of this study and should be explored in future research.

The results presented provide an important bridge between studies focusing on the spread of misinformation within online social media and those examining the propagation of fake news in society more broadly. It was also shown that the fragmentation of online social networks influences the average opinion of individuals within the same town—a result that was overlooked in previous research on this topic [36,65].

However, the findings do not control for active engagement with COVID-19 news. While the study analysis controls for household experience with COVID-19, institutional trust, and fear of COVID-19—which may partially account for active news consumption and abstention—there is evidence of personal network effects on news abstention related to COVID-19 [66]. Part of the results may thus reflect network effects on abstention; that is, in certain networks, individuals may be less willing to discuss specific topics. The study hypothesized that such situations are more likely in smaller networks, where each tie holds relatively greater value. However, the study findings show that network size, if anything, is negatively associated with the popularity of fake statements. This suggests that people in smaller towns are not necessarily more likely to abstain from discussing COVID-19.

### Limitations

This study had several limitations, which the author attempted to address and clarify in terms of their potential impact on the results. First, the network data were collected in 2023, whereas the survey was conducted in 2020. This time gap introduces a measurement error bias, which likely reduces the statistical significance of the results [67]. Additionally, the method used for sampling networks has limitations, as discussed in the “About VK” section. The study assumes that connections between accounts persist over time and that there has been no substantial drift in the fragmentation of towns—an assumption necessary for interpreting the observed correlations. Moreover, VK social networks are not representative of the entire population and do not capture all social interactions among Russian citizens. To estimate the potential bias introduced by using VK networks as a proxy for actual social interaction networks, demographic data from VK accounts and survey responses were compared. This information is provided in Multimedia Appendix 7. A similar age and gender structure was observed between the 2 groups, but there was a substantial difference in the proportion of individuals with higher education. The survey data likely overrepresent individuals with higher education compared with the general population. Additionally, users with higher education may not consistently disclose their educational background on VK, as this information is not required to use the platform. Importantly, this research does not claim a causal relationship between fragmentation and the spread of conspiracy theories.

To partially test the assumption of network structure stability, additional data in June 2023 and January 2024 (a 7- and 11-month gap, respectively) were collected. The results of the paired *t* test are presented in Multimedia Appendix 4. No significant differences in town-level fragmentation were found between 2023 and 2024. This suggests that network structure is either persistent over time or shaped by stable societal traits. While network scientists have increasingly focused on the emotional aspects of social ties, the formation of social ties and network structures remains understudied [68]. Social scientists often link network structure to generalized trust, a key component of social capital [62], and generalized trust is known to be stable over long periods [69]. Therefore, even if the 2023 fragmentation measure does not serve as a perfect proxy for actual fragmentation in 2020, it likely still captures elements of social capital that are persistent over time.

A robustness check was conducted using a subsample of towns with higher edge coverage. Previous research has indicated that an edge coverage of 15% yields a network sample that effectively preserves its structural characteristics [60]. This study analyzed a sample of 149 towns, excluding those with the lowest edge coverage, as they are more prone to bias. This adjustment increased the average edge coverage from 12.72% (688,859/5,416,402) to 15.04% (739,677/4,917,172). The results of these robustness checks are presented in Multimedia Appendix 6. The main outcomes discussed in this study remain robust even after excluding towns that may produce biased estimates of network variables.

### Conclusions

The results presented are particularly relevant in the context of the increasing spread of fake news, misinformation, and disinformation. They suggest that it is not only the sources of information that matter but also the structure of interpersonal networks. More broadly, these findings highlight the critical role of social networks in the dissemination of information.

This study demonstrates the indirect effects of online social media structures on the spread of both information and misinformation about COVID-19, as well as on changes in public attitudes. The results underscore the relative importance of the social networks individuals belong to and how information flows through these networks.

For future research, it is important to develop a clearer understanding of the mechanisms linking social media use, personal networks, and town-level modularity to fully capture the multilevel effects of social media on the spread of misinformation and attitudes toward it. Additionally, future studies should consider using more advanced methods of network sampling.

## Appendix

Multimedia Appendix 1 Parameters of the survey.

Multimedia Appendix 2 Information about municipal statistics data for the construction of control variables.

Multimedia Appendix 3 Information about towns in the sample.

Multimedia Appendix 4 Information about fragmentation index from different periods.

Multimedia Appendix 5 Additional information about network sampling.

Multimedia Appendix 6 Robustness checks. Using the dataset with over 15% average edge coverage.

Multimedia Appendix 7 Comparison of demographic information between accounts of Vkontakte (VK) users and respondents in the poll.

## Abbreviations

MTurk: Mechanical Turk
RoCIRR: Research on COVID-19 in Russia’s Regions
VK: Vkontakte
