# Supplementary material for: Modularity of Online Social Networks and COVID-19 Misinformation Spreading in Russia: Combining Social Network Analysis and National Representative Survey
Source: JMIR Infodemiology. 2025 Jun 26;5:e58302. doi: 10.2196/58302 (PMC12246759; doi:10.2196/58302)
Supplement: Multimedia Appendix 1 [file infodemiology_v5i1e58302_app1.docx]

Appendix 1. Parameters of the survey

The survey was divided into three parts: an OMI panel from towns with over 100’000 inhabitants, an additional sample from smaller towns and rural areas, and towns with less than 100’000 inhabitants.

The first part includes 16550 respondents from 39 regions. All respondents are from cities with over 100’000 inhabitants. Quotas for age, gender, and education were used to create samples.

The second part includes 5000 respondents from 22 regions. Respondents are not required to be from cities with over 100’000 inhabitants but most of them were from bigger towns. In this part, demographic quotas were less strict.

The third part includes 1270 respondents from 17 regions. Each region had at least 30 respondents and the same demographic quotas as in part two were introduced.

| Region Name | Number of observations | Share Female | Share in age between 18 and 24 | Share in age over 55 | Share without higher education degree |
| --- | --- | --- | --- | --- | --- |
| Altai krai | 280 | 0,567857 | 0,096429 | 0,053571 | 0,385714 |
| Arkhangelsk oblast | 196 | 0,668367 | 0,056122 | 0,02551 | 0,387755 |
| Astrakhan oblast | 172 | 0,715116 | 0,075581 | 0,017442 | 0,366279 |
| Bashkortostan | 466 | 0,562232 | 0,094421 | 0,034335 | 0,360515 |
| Belgorod oblast | 288 | 0,677083 | 0,097222 | 0,027778 | 0,361111 |
| Bryansk oblast | 205 | 0,697561 | 0,053659 | 0,019512 | 0,263415 |
| Chelyabinsk oblast | 481 | 0,594595 | 0,095634 | 0,033264 | 0,361746 |
| Irkutsk oblast | 285 | 0,585965 | 0,105263 | 0,045614 | 0,4 |
| Ivanovo oblast | 209 | 0,645933 | 0,062201 | 0,009569 | 0,397129 |
| Kaliningrad oblast | 176 | 0,653409 | 0,068182 | 0,039773 | 0,375 |
| Kaluga oblast | 136 | 0,698529 | 0,044118 | 0,036765 | 0,330882 |
| Karelia | 96 | 0,6875 | 0,083333 | 0,03125 | 0,3125 |
| Kemerovo oblast | 286 | 0,583916 | 0,104895 | 0,024476 | 0,388112 |
| Khabarovsk krai | 250 | 0,672 | 0,108 | 0,028 | 0,384 |
| Khanty-Mansi Autonomous Okrug | 138 | 0,594203 | 0,108696 | 0,043478 | 0,318841 |
| Kirov oblast | 280 | 0,689286 | 0,121429 | 0,046429 | 0,389286 |
| Komi Republic | 131 | 0,664122 | 0,099237 | 0,007634 | 0,389313 |
| Kostroma oblast | 134 | 0,686567 | 0,052239 | 0,059701 | 0,335821 |
| Krasnodar krai | 486 | 0,590535 | 0,104938 | 0,028807 | 0,374486 |
| Krasnoyarsk krai | 294 | 0,578231 | 0,105442 | 0,030612 | 0,37415 |
| Kurgan oblast | 134 | 0,649254 | 0,067164 | 0,022388 | 0,380597 |
| Kursk oblast | 183 | 0,704918 | 0,081967 | 0,027322 | 0,333333 |
| Leningrad oblast | 116 | 0,655172 | 0,077586 | 0,025862 | 0,318966 |
| Lipetsk oblast | 287 | 0,679443 | 0,111498 | 0,041812 | 0,372822 |
| Marij El | 93 | 0,698925 | 0,107527 | 0,032258 | 0,408602 |
| Mordovia | 129 | 0,620155 | 0,077519 | 0,023256 | 0,302326 |
| Moscow oblast | 262 | 0,572519 | 0,10687 | 0,038168 | 0,385496 |
| Murmansk oblast | 93 | 0,709677 | 0,064516 | 0,010753 | 0,376344 |
| Nizhni Novgorod oblast | 512 | 0,578125 | 0,105469 | 0,021484 | 0,404297 |
| Novgorod oblast | 98 | 0,663265 | 0,091837 | 0,020408 | 0,408163 |
| Novosibirsk oblast | 480 | 0,585417 | 0,089583 | 0,03125 | 0,375 |
| Omsk oblast | 482 | 0,576763 | 0,114108 | 0,039419 | 0,383817 |
| Orenburg oblast | 290 | 0,6 | 0,096552 | 0,048276 | 0,37931 |
| Oryol oblast | 100 | 0,71 | 0,06 | 0,01 | 0,28 |
| Penza oblast | 177 | 0,694915 | 0,056497 | 0,022599 | 0,310734 |
| Perm krai | 494 | 0,605263 | 0,109312 | 0,040486 | 0,388664 |
| Primorsky krai | 284 | 0,683099 | 0,105634 | 0,03169 | 0,387324 |
| Pskov oblast | 99 | 0,656566 | 0,050505 | 0,020202 | 0,323232 |
| Rostov oblast | 486 | 0,582305 | 0,104938 | 0,028807 | 0,382716 |
| Ryazan oblast | 265 | 0,656604 | 0,120755 | 0,033962 | 0,407547 |
| Saint-Petersburg | 437 | 0,558352 | 0,10984 | 0,043478 | 0,393593 |
| Samara oblast | 488 | 0,563525 | 0,098361 | 0,026639 | 0,389344 |
| Saratov oblast | 473 | 0,585624 | 0,097252 | 0,031712 | 0,372093 |
| Smolensk oblast | 161 | 0,664596 | 0,049689 | 0,031056 | 0,329193 |
| Stavropol krai | 280 | 0,592857 | 0,107143 | 0,05 | 0,382143 |
| Sverdlovsk oblast | 505 | 0,576238 | 0,106931 | 0,031683 | 0,4 |
| Tambov oblast | 122 | 0,639344 | 0,07377 | 0,02459 | 0,368852 |
| Tatarstan | 479 | 0,578288 | 0,114823 | 0,029228 | 0,40501 |
| The Chuvash Republic | 288 | 0,65625 | 0,104167 | 0,034722 | 0,395833 |
| Tomsk oblast | 261 | 0,659004 | 0,118774 | 0,042146 | 0,413793 |
| Tula oblast | 288 | 0,666667 | 0,104167 | 0,034722 | 0,381944 |
| Tver oblast | 181 | 0,701657 | 0,049724 | 0,022099 | 0,342541 |
| Tyumen oblast | 154 | 0,564935 | 0,12987 | 0,038961 | 0,461039 |
| Udmurtia | 291 | 0,642612 | 0,116838 | 0,041237 | 0,388316 |
| Ulyanovsk oblast | 304 | 0,674342 | 0,101974 | 0,042763 | 0,394737 |
| Vladimir oblast | 277 | 0,696751 | 0,122744 | 0,032491 | 0,386282 |
| Volgograd oblast | 472 | 0,601695 | 0,103814 | 0,040254 | 0,385593 |
| Vologda oblast | 271 | 0,682657 | 0,099631 | 0,04059 | 0,391144 |
| Voronezh oblast | 503 | 0,584493 | 0,119284 | 0,037773 | 0,38171 |
| Yaroslavl oblast | 299 | 0,602007 | 0,113712 | 0,033445 | 0,391304 |

Appendix table 3. Regions and parameters of the survey
