# Supplementary material for: Modularity of Online Social Networks and COVID-19 Misinformation Spreading in Russia: Combining Social Network Analysis and National Representative Survey
Source: JMIR Infodemiology. 2025 Jun 26;5:e58302. doi: 10.2196/58302 (PMC12246759; doi:10.2196/58302)
Supplement: Multimedia Appendix 2 [file infodemiology_v5i1e58302_app2.docx]

Appendix. 2. Municipal statistics

To construct town level variables of wages and town population multiple sources of data within Rosstat were used. Data about towns are usually not presented by the Rosstat except for the list of biggest cities, such information is published as part of yearly reports about Russian regions. For other towns, data was collected by using municipal statistics.

To construct an average yearly wage municipality was found in the list of municipalities.

If a town was itself a municipality and it was the head of an administrative unit usually wages were published for the whole administrative unit but not the town itself. In such situations, wages were taken from the administrative unit.

Wages were transformed from nominal terms into real ones by using town level and regional level price indexes of Rosstat if town level were not present.

Population statistics were also taken from town level statistics for bigger towns and from municipal statistics for smaller ones. All statistics were transformed into the same units for comparison.
