# Supplementary material for: Modularity of Online Social Networks and COVID-19 Misinformation Spreading in Russia: Combining Social Network Analysis and National Representative Survey
Source: JMIR Infodemiology. 2025 Jun 26;5:e58302. doi: 10.2196/58302 (PMC12246759; doi:10.2196/58302)
Supplement: Multimedia Appendix 3 [file infodemiology_v5i1e58302_app3.docx]

Appendix 3.

| Region | Town | Number of respondents | Number of nodes | Population, 2019 | Share of VK users | Number of nodes in the network to population | Nodes to population adjusted to share of VK users |
| --- | --- | --- | --- | --- | --- | --- | --- |
| Rostov oblast | Azov | 9 | 31363 | 80286 | 0,667 | 0,391 | 0,586 |
| Tatarstan | Almetyevsk | 18 | 87476 | 157310 | 0,722 | 0,556 | 0,77 |
| Irkutsk oblast | Angarsk | 58 | 53376 | 224600 | 0,517 | 0,238 | 0,459 |
| Murmansk oblast | Apatity | 10 | 31312 | 55201 | 0,7 | 0,567 | 0,81 |
| Nizhni Novgorod oblast | Arzamas | 17 | 61699 | 104000 | 0,588 | 0,593 | 1,009 |
| Krasnodar krai | Armavir | 17 | 45236 | 207600 | 0,471 | 0,218 | 0,463 |
| Primorsky krai | Artem | 13 | 11442 | 106460 | 0,308 | 0,107 | 0,349 |
| Arkhangelsk oblast | Arkhangelsk | 139 | 163338 | 354100 | 0,676 | 0,461 | 0,682 |
| Astrakhan oblast | Astrakhan | 172 | 12106 | 529800 | 0,541 | 0,023 | 0,042 |
| Krasnoyarsk krai | Achinsk | 20 | 49349 | 106800 | 0,4 | 0,462 | 1,155 |
| Saratov oblast | Balakovo | 48 | 51140 | 187500 | 0,688 | 0,273 | 0,397 |
| Moscow oblast | Balashikha | 34 | 35661 | 490047 | 0,5 | 0,073 | 0,146 |
| Altai krai | Barnaul | 205 | 109547 | 697000 | 0,546 | 0,157 | 0,288 |
| Rostov oblast | Bataysk | 12 | 15118 | 127654 | 0,5 | 0,118 | 0,237 |
| Belgorod oblast | Belgorod | 191 | 181066 | 394100 | 0,654 | 0,459 | 0,702 |
| Novosibirsk oblast | Berdsk | 15 | 26772 | 104300 | 0,667 | 0,257 | 0,385 |
| Perm krai | Berezniki | 21 | 55015 | 153200 | 0,81 | 0,359 | 0,444 |
| Altai krai | Biysk | 54 | 65493 | 209200 | 0,519 | 0,313 | 0,604 |
| Novgorod oblast | Borovichi | 9 | 33141 | 49071 | 0,667 | 0,675 | 1,013 |
| Irkutsk oblast | Bratsk | 41 | 56353 | 226300 | 0,463 | 0,249 | 0,537 |
| Bryansk oblast | Bryansk | 183 | 102236 | 420400 | 0,683 | 0,243 | 0,356 |
| Pskov oblast | Velikie Luki | 22 | 59359 | 90549 | 0,909 | 0,656 | 0,721 |
| Novgorod oblast | Velikiy Novgorod | 89 | 115289 | 224900 | 0,787 | 0,513 | 0,652 |
| Primorsky krai | Vladivostok | 201 | 42604 | 634700 | 0,388 | 0,067 | 0,173 |
| Vladimir oblast | Vladimir | 192 | 74520 | 359400 | 0,646 | 0,207 | 0,321 |
| Volgograd oblast | Volgograd | 377 | 107862 | 1009000 | 0,512 | 0,107 | 0,209 |
| Rostov oblast | Volgodonsk | 16 | 52107 | 171400 | 0,375 | 0,304 | 0,811 |
| Volgograd oblast | Volzhsky | 79 | 54289 | 323604 | 0,595 | 0,168 | 0,282 |
| Vologda oblast | Vologda | 177 | 146317 | 310300 | 0,734 | 0,472 | 0,642 |
| Leningrad oblast | Volkhov | 12 | 16767 | 44487 | 0,667 | 0,377 | 0,565 |
| Komi Republic | Vorkuta | 11 | 34054 | 54223 | 0,636 | 0,628 | 0,987 |
| Voronezh oblast | Voronezh | 495 | 94228 | 1058300 | 0,602 | 0,089 | 0,148 |
| Leningrad oblast | Vsevolozhsk | 10 | 7376 | 74263 | 0,8 | 0,099 | 0,124 |
| Leningrad oblast | Vyborg | 21 | 44797 | 76389 | 0,81 | 0,586 | 0,724 |
| Smolensk oblast | Vyazma | 12 | 19897 | 51790 | 0,833 | 0,384 | 0,461 |
| Leningrad oblast | Gatchina | 17 | 33931 | 93710 | 0,765 | 0,362 | 0,473 |
| Belgorod oblast | Gubkin | 8 | 24804 | 86422 | 0,5 | 0,287 | 0,574 |
| Nizhni Novgorod oblast | Dzerzhinsk | 48 | 84612 | 238800 | 0,771 | 0,354 | 0,46 |
| Ulyanovsk oblast | Dimitrovgrad | 27 | 55159 | 113500 | 0,593 | 0,486 | 0,82 |
| Moscow oblast | Domodedovo | 11 | 30589 | 133528 | 0,727 | 0,229 | 0,315 |
| Sverdlovsk oblast | Ekaterinburg | 434 | 88408 | 1526400 | 0,583 | 0,058 | 0,099 |
| Lipetsk oblast | Elets | 35 | 38453 | 102300 | 0,514 | 0,376 | 0,731 |
| Stavropol krai | Essentuki | 22 | 27005 | 110479 | 0,409 | 0,244 | 0,598 |
| Kursk oblast | Zheleznogorsk | 12 | 32019 | 100400 | 0,667 | 0,319 | 0,478 |
| Moscow oblast | Zhukovsky | 10 | 23100 | 107922 | 0,8 | 0,214 | 0,268 |
| Chelyabinsk oblast | Zlatoust | 16 | 63860 | 165375 | 0,625 | 0,386 | 0,618 |
| Ivanovo oblast | Ivanovo | 190 | 114681 | 404600 | 0,637 | 0,283 | 0,445 |
| Udmurtia | Izhevsk | 291 | 233141 | 648100 | 0,687 | 0,36 | 0,523 |
| Irkutsk oblast | Irkutsk | 186 | 193556 | 623600 | 0,473 | 0,31 | 0,656 |
| Marij El | Yoshkar-Ola | 93 | 119651 | 285500 | 0,656 | 0,419 | 0,639 |
| Tatarstan | Kazan | 354 | 362871 | 1257400 | 0,619 | 0,289 | 0,466 |
| Kaliningrad oblast | Kaliningrad | 176 | 80294 | 489300 | 0,597 | 0,164 | 0,275 |
| Kaluga oblast | Kaluga | 114 | 110954 | 332000 | 0,649 | 0,334 | 0,515 |
| Sverdlovsk oblast | Kamensk-Uralsky | 15 | 55742 | 167800 | 0,6 | 0,332 | 0,554 |
| Volgograd oblast | Kamyshin | 16 | 29663 | 109900 | 0,438 | 0,27 | 0,617 |
| Kemerovo oblast | Kemerovo | 153 | 66269 | 556400 | 0,66 | 0,119 | 0,18 |
| Ivanovo oblast | Kineshma | 10 | 19453 | 81986 | 0,6 | 0,237 | 0,395 |
| Leningrad oblast | Kirishi | 8 | 25650 | 50750 | 0,75 | 0,505 | 0,674 |
| Kirov oblast | Kirov | 280 | 140113 | 543800 | 0,768 | 0,258 | 0,336 |
| Stavropol krai | Kislovodsk | 11 | 20794 | 129098 | 0,364 | 0,161 | 0,443 |
| Bryansk oblast | Klintsy | 11 | 20051 | 63050 | 0,455 | 0,318 | 0,7 |
| Vladimir oblast | Kovrov | 44 | 49260 | 135700 | 0,636 | 0,363 | 0,57 |
| Moscow oblast | Kolomna | 10 | 55871 | 141106 | 0,5 | 0,396 | 0,792 |
| Khabarovsk krai | Komsomolsk-on-Amur | 46 | 59545 | 244800 | 0,478 | 0,243 | 0,509 |
| Moscow oblast | Korolev | 10 | 28511 | 224533 | 0,4 | 0,127 | 0,317 |
| Arkhangelsk oblast | Koryazhma | 13 | 23857 | 36224 | 0,692 | 0,659 | 0,951 |
| Kostroma oblast | Kostroma | 134 | 82094 | 276900 | 0,59 | 0,296 | 0,503 |
| Arkhangelsk oblast | Kotlas | 7 | 40356 | 61821 | 1 | 0,653 | 0,653 |
| Moscow oblast | Krasnogorsk | 14 | 20943 | 175600 | 0,571 | 0,119 | 0,209 |
| Krasnodar krai | Krasnodar | 364 | 52251 | 1022000 | 0,547 | 0,051 | 0,094 |
| Krasnoyarsk krai | Krasnoyarsk | 259 | 229070 | 1094500 | 0,583 | 0,209 | 0,359 |
| Penza oblast | Kuznetsk | 10 | 29176 | 81027 | 0,8 | 0,36 | 0,45 |
| Perm krai | Kungur | 10 | 39095 | 65284 | 0,8 | 0,599 | 0,749 |
| Kurgan oblast | Mound | 120 | 113549 | 312400 | 0,7 | 0,363 | 0,519 |
| Kursk oblast | Kursk | 171 | 154173 | 453000 | 0,649 | 0,34 | 0,524 |
| Lipetsk oblast | Lipetsk | 252 | 156967 | 508600 | 0,667 | 0,309 | 0,463 |
| Perm krai | Lysva | 10 | 23370 | 60947 | 0,8 | 0,383 | 0,479 |
| Moscow oblast | Lyubertsy | 20 | 10583 | 205300 | 0,6 | 0,052 | 0,086 |
| Chelyabinsk oblast | Magnitogorsk | 72 | 170342 | 413300 | 0,708 | 0,412 | 0,582 |
| Chelyabinsk oblast | Miass | 21 | 66988 | 166400 | 0,619 | 0,403 | 0,65 |
| Moscow oblast | Moscow | 41 | 541123 | 12615279 | 0,707 | 0,043 | 0,061 |
| Murmansk oblast | Murmansk | 73 | 125928 | 287800 | 0,836 | 0,438 | 0,524 |
| Vladimir oblast | Murom | 41 | 39615 | 115600 | 0,585 | 0,343 | 0,585 |
| Moscow oblast | Mytishchi | 13 | 26504 | 222739 | 0,615 | 0,119 | 0,193 |
| Tatarstan | Naberezhnye Chelny | 83 | 154720 | 533800 | 0,59 | 0,29 | 0,491 |
| Primorsky krai | Nakhodka | 25 | 33728 | 146000 | 0,2 | 0,231 | 1,155 |
| Stavropol krai | Nevinnomyssk | 27 | 25179 | 116700 | 0,593 | 0,216 | 0,364 |
| Bashkortostan | Neftekamsk | 14 | 69555 | 131100 | 0,429 | 0,531 | 1,238 |
| Khanty-Mansi Autonomous Okrug | Nefteyugansk | 13 | 39475 | 127710 | 0,615 | 0,309 | 0,502 |
| Khanty-Mansi Autonomous Okrug | Nizhnevartovsk | 49 | 91846 | 277700 | 0,592 | 0,331 | 0,559 |
| Tatarstan | Nizhnekamsk | 24 | 98838 | 238879 | 0,75 | 0,414 | 0,552 |
| Nizhni Novgorod oblast | Nizhny Novgorod | 447 | 201525 | 1271800 | 0,64 | 0,158 | 0,248 |
| Sverdlovsk oblast | Nizhny Tagil | 45 | 122987 | 351600 | 0,733 | 0,35 | 0,477 |
| Bryansk oblast | Novozybkov | 11 | 12166 | 39725 | 0,818 | 0,306 | 0,374 |
| Kemerovo oblast | Novokuznetsk | 113 | 126473 | 549400 | 0,602 | 0,23 | 0,383 |
| Tula oblast | Novomoskovsk | 40 | 42501 | 133800 | 0,5 | 0,318 | 0,635 |
| Krasnodar krai | Novorossiysk | 33 | 92510 | 338800 | 0,455 | 0,273 | 0,601 |
| Novosibirsk oblast | Novosibirsk | 465 | 198065 | 1625600 | 0,574 | 0,122 | 0,212 |
| The Chuvash Republic | Novocheboksarsk | 39 | 30441 | 126794 | 0,744 | 0,24 | 0,323 |
| Rostov oblast | Novocherkassk | 27 | 41180 | 167355 | 0,407 | 0,246 | 0,604 |
| Moscow oblast | Noginsk | 7 | 33711 | 104000 | 0,714 | 0,324 | 0,454 |
| Krasnoyarsk krai | Norilsk | 15 | 47842 | 182500 | 0,467 | 0,262 | 0,562 |
| Kaluga oblast | Obninsk | 22 | 35330 | 117400 | 0,636 | 0,301 | 0,473 |
| Bashkortostan | Oktyabrskiy | 14 | 41664 | 114194 | 0,643 | 0,365 | 0,568 |
| Omsk oblast | Omsk | 482 | 253254 | 1154500 | 0,618 | 0,219 | 0,355 |
| Oryol oblast | Oryol | 100 | 83892 | 311625 | 0,72 | 0,269 | 0,374 |
| Orenburg oblast | Orenburg | 238 | 181601 | 587000 | 0,605 | 0,309 | 0,511 |
| Moscow oblast | Orekhovo-Zuevo | 15 | 41961 | 118300 | 0,533 | 0,355 | 0,665 |
| Orenburg oblast | Orsk | 52 | 63782 | 230400 | 0,692 | 0,277 | 0,4 |
| Penza oblast | Penza | 167 | 180573 | 520300 | 0,599 | 0,347 | 0,58 |
| Sverdlovsk oblast | Pervouralsk | 11 | 44509 | 143100 | 0,818 | 0,311 | 0,38 |
| Perm krai | Perm | 453 | 289712 | 1055400 | 0,746 | 0,275 | 0,368 |
| Karelia | Petrozavodsk | 96 | 136552 | 281000 | 0,76 | 0,486 | 0,639 |
| Moscow oblast | Podolsk | 17 | 62749 | 308100 | 0,647 | 0,204 | 0,315 |
| Kemerovo oblast | Prokopyevsk | 20 | 56490 | 190300 | 0,7 | 0,297 | 0,424 |
| Pskov oblast | Pskov | 77 | 124356 | 210300 | 0,74 | 0,591 | 0,799 |
| Stavropol krai | Pyatigorsk | 35 | 63477 | 214600 | 0,429 | 0,296 | 0,69 |
| Tver oblast | Rzhev | 10 | 24590 | 58596 | 0,7 | 0,42 | 0,6 |
| Voronezh oblast | Rossosh | 8 | 20628 | 62716 | 0,625 | 0,329 | 0,526 |
| Rostov oblast | Rostov-on-Don | 348 | 168199 | 1137900 | 0,546 | 0,148 | 0,271 |
| Altai krai | Rubtsovsk | 21 | 31926 | 141600 | 0,238 | 0,225 | 0,947 |
| Yaroslavl oblast | Rybinsk | 37 | 97395 | 184600 | 0,757 | 0,528 | 0,697 |
| Ryazan oblast | Ryazan | 265 | 148917 | 539300 | 0,683 | 0,276 | 0,404 |
| Bashkortostan | Salavat | 18 | 57268 | 151571 | 0,722 | 0,378 | 0,523 |
| Samara oblast | Samara | 312 | 317236 | 1156700 | 0,635 | 0,274 | 0,432 |
| Leningrad oblast | Saint Petersburg | 462 | 422346 | 5383890 | 0,61 | 0,078 | 0,129 |
| Mordovia | Saransk | 129 | 129410 | 349800 | 0,698 | 0,37 | 0,53 |
| Saratov oblast | Saratov | 381 | 136321 | 838000 | 0,538 | 0,163 | 0,302 |
| Arkhangelsk oblast | Severodvinsk | 37 | 96024 | 183000 | 0,73 | 0,525 | 0,719 |
| Murmansk oblast | Severomorsk | 10 | 16090 | 52597 | 0,8 | 0,306 | 0,382 |
| Tomsk oblast | Seversk | 31 | 22508 | 107036 | 0,484 | 0,21 | 0,435 |
| Moscow oblast | Sergiev Posad | 9 | 45537 | 100300 | 0,778 | 0,454 | 0,584 |
| Moscow oblast | Serpukhov | 11 | 46294 | 126300 | 0,727 | 0,367 | 0,504 |
| Smolensk oblast | Smolensk | 149 | 113858 | 325500 | 0,604 | 0,35 | 0,579 |
| Leningrad oblast | Sosnovyj Bor | 11 | 15779 | 68344 | 0,545 | 0,231 | 0,423 |
| Krasnodar krai | Sochi | 72 | 375 | 530400 | 0,431 | 0,001 | 0,002 |
| Stavropol krai | Stavropol | 185 | 101800 | 450900 | 0,497 | 0,226 | 0,454 |
| Belgorod oblast | Stary Oskol | 89 | 85141 | 223900 | 0,652 | 0,38 | 0,584 |
| Bashkortostan | Sterlitamak | 44 | 110120 | 276400 | 0,705 | 0,398 | 0,565 |
| Khanty-Mansi Autonomous Okrug | Surgut | 76 | 145597 | 380600 | 0,553 | 0,383 | 0,692 |
| Samara oblast | Sizran | 28 | 61170 | 167800 | 0,679 | 0,365 | 0,537 |
| Komi Republic | Syktyvkar | 97 | 132898 | 259900 | 0,711 | 0,511 | 0,719 |
| Rostov oblast | Taganrog | 51 | 85157 | 248600 | 0,471 | 0,343 | 0,728 |
| Tambov oblast | Tambov | 122 | 131853 | 292100 | 0,59 | 0,451 | 0,765 |
| Tver oblast | Tver | 171 | 159136 | 425100 | 0,684 | 0,374 | 0,547 |
| Leningrad oblast | Tikhvin | 12 | 34682 | 58068 | 0,833 | 0,597 | 0,717 |
| Samara oblast | Tolyatti | 148 | 177654 | 699400 | 0,716 | 0,254 | 0,355 |
| Tomsk oblast | Tomsk | 230 | 158168 | 597800 | 0,622 | 0,265 | 0,426 |
| Tula oblast | Tula | 248 | 100162 | 542500 | 0,552 | 0,185 | 0,334 |
| Tyumen oblast | Tyumen | 154 | 296816 | 807300 | 0,623 | 0,368 | 0,59 |
| Ulyanovsk oblast | Ulyanovsk | 277 | 137973 | 650300 | 0,646 | 0,212 | 0,328 |
| Primorsky krai | Ussuriysk | 45 | 38626 | 199300 | 0,444 | 0,194 | 0,436 |
| Bashkortostan | Ufa | 376 | 297359 | 1128800 | 0,628 | 0,263 | 0,42 |
| Komi Republic | Ukhta | 23 | 55336 | 113700 | 0,609 | 0,487 | 0,8 |
| Khabarovsk krai | Khabarovsk | 204 | 97523 | 616400 | 0,5 | 0,158 | 0,316 |
| Moscow oblast | Khimki | 14 | 8797 | 254748 | 0,429 | 0,035 | 0,081 |
| The Chuvash Republic | Cheboksary | 249 | 154158 | 508100 | 0,618 | 0,303 | 0,491 |
| Chelyabinsk oblast | Chelyabinsk | 372 | 202920 | 1196700 | 0,637 | 0,17 | 0,266 |
| Vologda oblast | Cherepovets | 94 | 160862 | 314800 | 0,702 | 0,511 | 0,728 |
| Kurgan oblast | Shadrinsk | 14 | 32283 | 74929 | 0,643 | 0,431 | 0,67 |
| Rostov oblast | Shakhti | 23 | 45912 | 230300 | 0,478 | 0,199 | 0,417 |
| Ivanovo oblast | Shuya | 9 | 20350 | 57569 | 0,444 | 0,353 | 0,795 |
| Moscow oblast | Shchelkovo | 16 | 22696 | 124831 | 0,563 | 0,182 | 0,323 |
| Moscow oblast | Elektrostal | 10 | 38046 | 156000 | 0,8 | 0,244 | 0,305 |
| Saratov oblast | Engels | 44 | 35050 | 225731 | 0,614 | 0,155 | 0,253 |
| Yaroslavl oblast | Yaroslavl | 262 | 189177 | 608400 | 0,653 | 0,311 | 0,476 |

Appendix table 1. Network data of Russian towns

| Population | number of towns in the sample |
| --- | --- |
| 100000 | 27 |
| 250000 | 68 |
| 499999 | 32 |
| 500000 | 39 |

Appendix table 1.1. Distribution of towns in the sample by population
