# Supplementary material for: Modularity of Online Social Networks and COVID-19 Misinformation Spreading in Russia: Combining Social Network Analysis and National Representative Survey
Source: JMIR Infodemiology. 2025 Jun 26;5:e58302. doi: 10.2196/58302 (PMC12246759; doi:10.2196/58302)
Supplement: Multimedia Appendix 4 [file infodemiology_v5i1e58302_app4.docx]

To test whether there are significant differences between fragmentation indexes in 2023 and 2024 we used two-sided paired t-test.

Results of two-sided paired t-test:

t = 1.8468, df = 165, p-value = 0.06657

alternative hypothesis: true mean difference is not equal to 0

95 percent confidence interval: (-0.00096; 0.029)

Sample estimates: mean difference 0.01387926

We observe that difference is statistically indistinguishable from zero on 5% confidence level.

Moreover, some variation of fragmentation index is caused by heuristic nature of community finding algorithms. In Table 1 the estimates for different fragmentation indexes constructed from the same data are presented:

|  | mean | sd |
| --- | --- | --- |
| 2023 fragmentation index, recalculated mean | 0,4380387 | 0,08112065 |
| 2023 fragmentation index, recalculated 1 | 0,43628119 | 0,08072898 |
| 2023 fragmentation index, recalculated 2 | 0,44015259 | 0,08322235 |
| 2023fragmentation index, recalculated 3 | 0,43744013 | 0,08099962 |
| 2023 fragmentation index, recalculated 4 | 0,43375368 | 0,08029493 |
| 2023 fragmentation index, recalculated 5 | 0,44022163 | 0,08174331 |
| 2023 fragmentation index, recalculated 6 | 0,43621578 | 0,08251982 |
| 2023 fragmentation index, recalculated 7 | 0,4345655 | 0,08227757 |
| 2023 fragmentation index, recalculated 8 | 0,44008842 | 0,07861094 |
| 2023 fragmentation index, recalculated 9 | 0,43609883 | 0,08321792 |
| 2023 fragmentation index, recalculated 10 | 0,43939082 | 0,08061109 |
| 2023 fragmentation index, recalculated 11 | 0,44125843 | 0,0788589 |
| 2023 fragmentation index, recalculated 12 | 0,44106034 | 0,08135416 |
| 2023 fragmentation index, recalculated 13 | 0,43683302 | 0,08269489 |
| 2023 fragmentation index, recalculated 14 | 0,43989947 | 0,08 |
| 2023 fragmentation index, recalculated 15 | 0,43769329 | 0,08026549 |
| 2023 fragmentation index, recalculated 16 | 0,43879337 | 0,08181417 |
| 2023 fragmentation index, recalculated 17 | 0,44038398 | 0,08196027 |
| 2023 fragmentation index, recalculated 18 | 0,43626189 | 0,08171654 |
| 2023 fragmentation index, recalculated 19 | 0,43904043 | 0,08339592 |
| 2023 fragmentation index, recalculated 20 | 0,44015798 | 0,08065955 |
| 2023 fragmentation index, recalculated 21 | 0,43556448 | 0,08091777 |
| 2023 fragmentation index, recalculated 22 | 0,43595942 | 0,08398275 |
| 2023 fragmentation index, recalculated 23 | 0,4355688 | 0,08104134 |
| 2023 fragmentation index, recalculated 24 | 0,44054079 | 0,08035969 |
| 2023 fragmentation index, recalculated 25 | 0,43894157 | 0,08083218 |

Table 1. Fragmentation indexes constructed from the same data distributions.

|  | mean | sd |
| --- | --- | --- |
| 2023 fragmentation index, recalculated mean | 0,4380387 | 0,08112065 |
| 2023 fragmentation index | 0,4495772 | 0,09481117 |
| 2024 fragmentation index | 0,43492819 | 0,08000746 |

Table 2. Fragmentation indexes distributions.
