# Supplementary material for: Modularity of Online Social Networks and COVID-19 Misinformation Spreading in Russia: Combining Social Network Analysis and National Representative Survey
Source: JMIR Infodemiology. 2025 Jun 26;5:e58302. doi: 10.2196/58302 (PMC12246759; doi:10.2196/58302)
Supplement: Multimedia Appendix 5 [file infodemiology_v5i1e58302_app5.docx]

**Appendix 5. Network Sampling.**

The following part of the study is organized into two sections: first, the estimation of edge coverage for the final sample; second, an empirical assessment of the outcomes resulting from increasing the number of accounts sampled in the final network.

1. **Edge coverage**

To estimate the edge coverage of unknown networks, we must consider the degree of the ego networks of citizens. To derive the lower limit of edge coverage, we propose using the Dunbar number (which is approximately 100 people). By multiplying the number of citizens by half of the Dunbar number, we can obtain the expected number of edges. This allows us to calculate a coverage ratio by comparing the average number of edges in the network to the expected number of edges. Our findings indicate an edge coverage of 3.16% for the actual population of Russian towns. Additional relevant information can be found in Appendix Table 5.1.

| The average number of citizens | Expected number of edges | The average number of edges in the network | Coverage ratio |
| --- | --- | --- | --- |
| 435663 | 21783150 | 688859 | 3,16% |

Appendix Table 5.1. Lower limit of edge coverage ratio

To estimate the upper limit of edge coverage, we need to consider both the percentage of the population that uses VK and the proportion of friends in the friend lists who are from the same town. According to data from the RoCIRR poll, an average of 61.7% of respondents use VK. Additionally, based on a sample of 1,200 random accounts from selected towns, we found that the average percentage of friends from the same town is 40.3%. Using this information, we calculate the expected degree to be 24.87 and the edge coverage ratio to be 12.71%. Further details can be found in Appendix Table 5.2.

| Expected number of edges of VK users | Expected number of edges of VK users from the same town | The expected degree | Coverage ratio |
| --- | --- | --- | --- |
| 13440203,55 | 5416402,03 | 24,87 | 12,71% |

Appendix Table 5.2. Upper limit of edge coverage ratio

1. **Number of accounts selected to construct the network and characteristics of the networks**

To minimize potential bias introduced by the sampling strategy, we select up to 18 accounts to sample our final network for each town. In this section of the appendix, we present arguments demonstrating the adequacy of the number of selected accounts.

As it was explained in the text, each random account produces a network – a network of friends of such account and the network of friends of friends. To estimate the relationship between fragmentation index and the increase in the number of networks combined we use a dataset that iteratively combined networks and recalculated each index for 49 towns on data that was collected in 2023. In more recent network datasets, we first combine all networks in one network and then calculate network indexes which is more efficient.

| Number of networks connected to create the dataset | Number of towns |
| --- | --- |
| 10 | 2 |
| 12 | 1 |
| 13 | 1 |
| 14 | 4 |
| 15 | 8 |
| 16 | 7 |
| 17 | 7 |
| 18 | 19 |

Appendix Table 5.3. Distribution of towns in the sample by the number of networks combined

Overall, we observe that joining multiple networks affects the change fragmentation index and there is a negative association between these factors (Appendix Plot 5.1). However, we also observe that this relationship is driven by earlier iterations that increase the number of edges several times. As the number of networks connected increases the number of new edges introduced and the relationship with change in fragmentation index becomes insignificant or changes its sign.


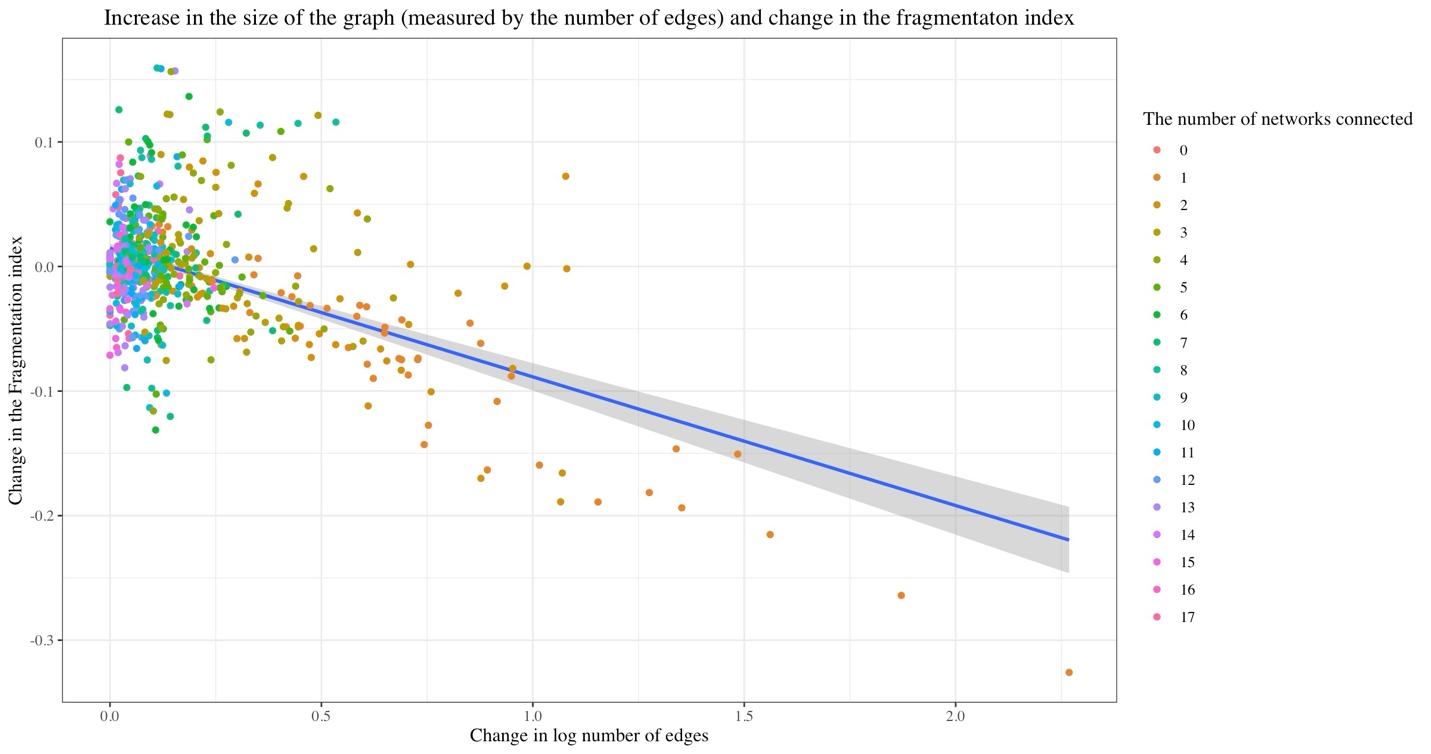


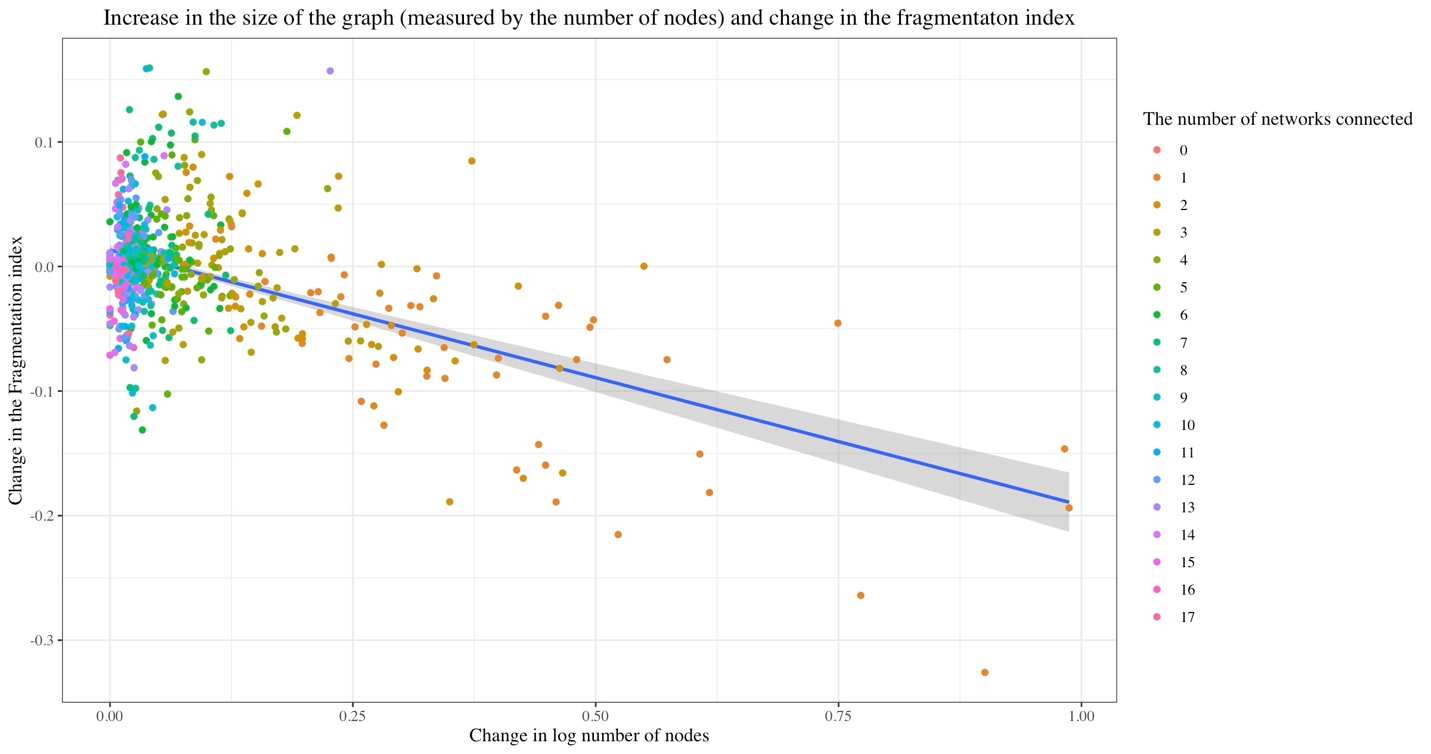


Appendix Plot 5.1. Network size change and change in the fragmentation index.

If we look at the results separately for each interaction, we observe that only huge spikes in the number of edges or nodes lead to significant changes in fragmentation (Appendix plot 5.2). We observe that such spikes happen after adding second and third graphs. We also observe significant spikes after adding 9 graphs and 14 graphs together.

Finally, we estimate confidence intervals of the average change in the log number of nodes and log number of edges by number of networks connected (Appendix plot 5.3). We observe that the number of nodes added decreases with the number of accounts selected, it does not change after joining over 10 networks together. We plot confidence intervals for the variance of the fragmentation index in Appendix Plot 5.4. We observe similar results – the variance of the fragmentation index falls dramatically after combining the first networks. We also observe higher variance in 18 combined networks, which is to be expected with the decrease in the number of networks in the sample.


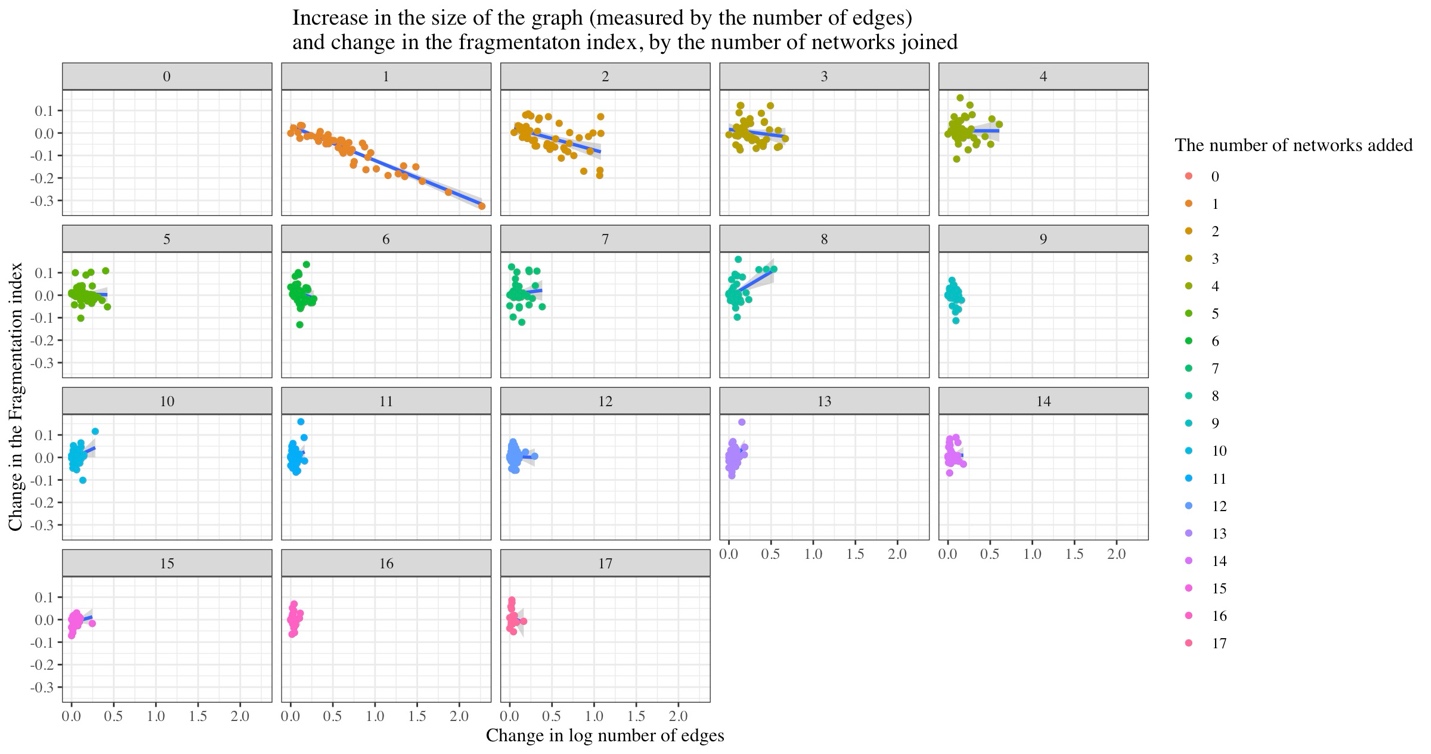


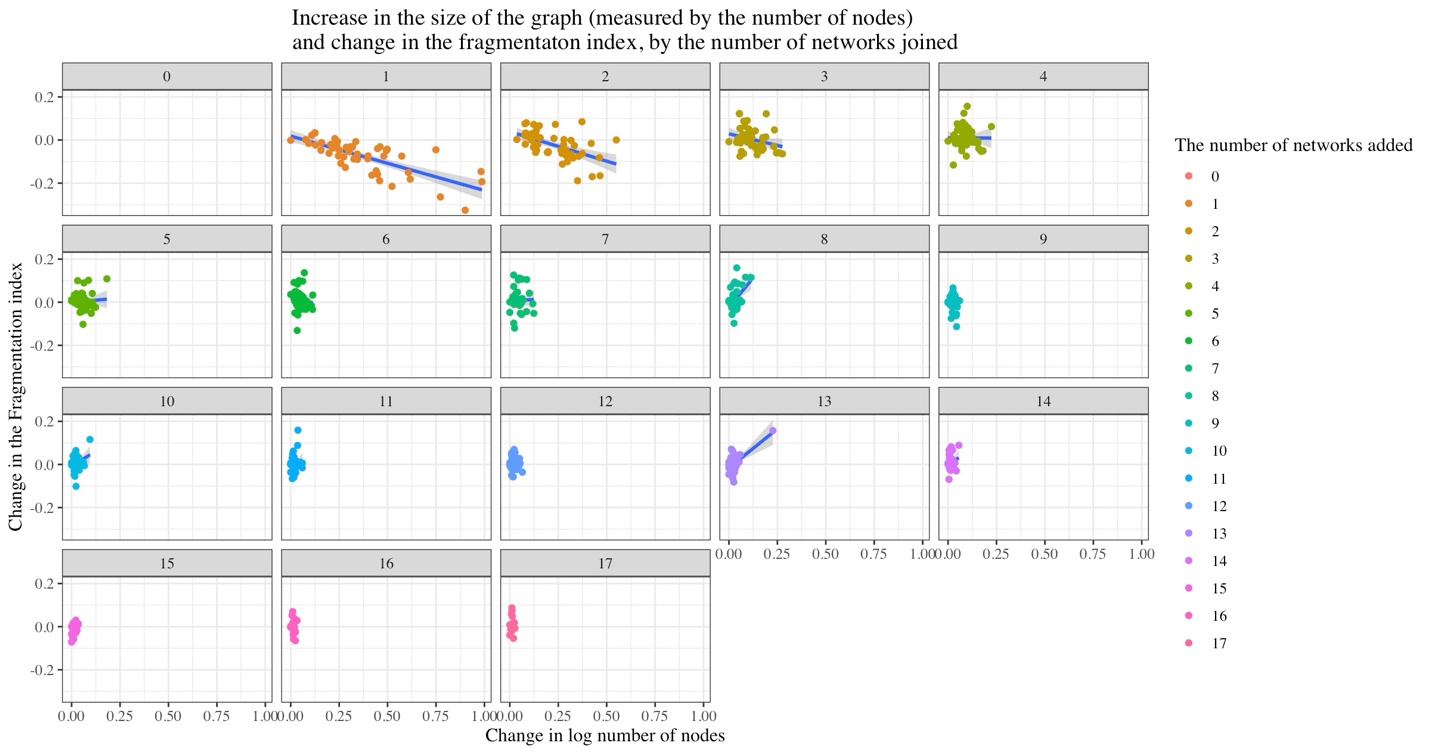


Appendix Plot 5.2. Network size change and change in the fragmentation index. Each iteration separately.


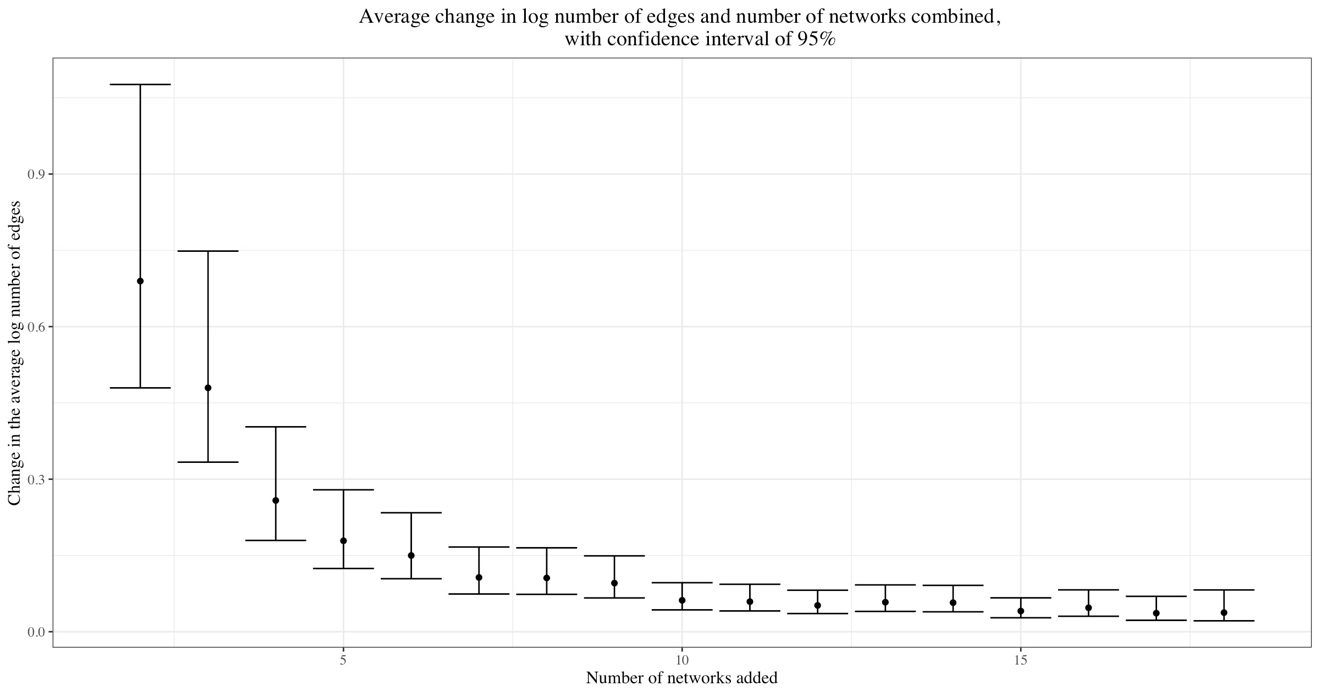


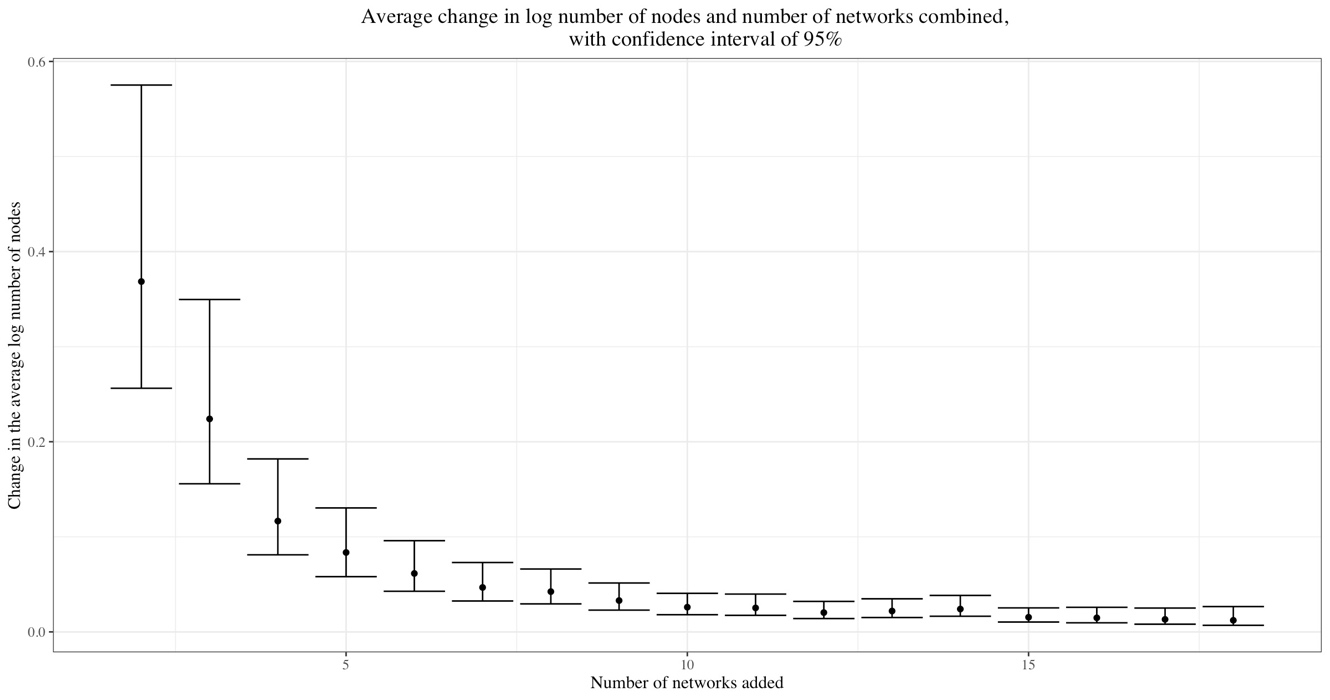


Appendix Plot 5.3. Number of networks added and change in the size of the network.


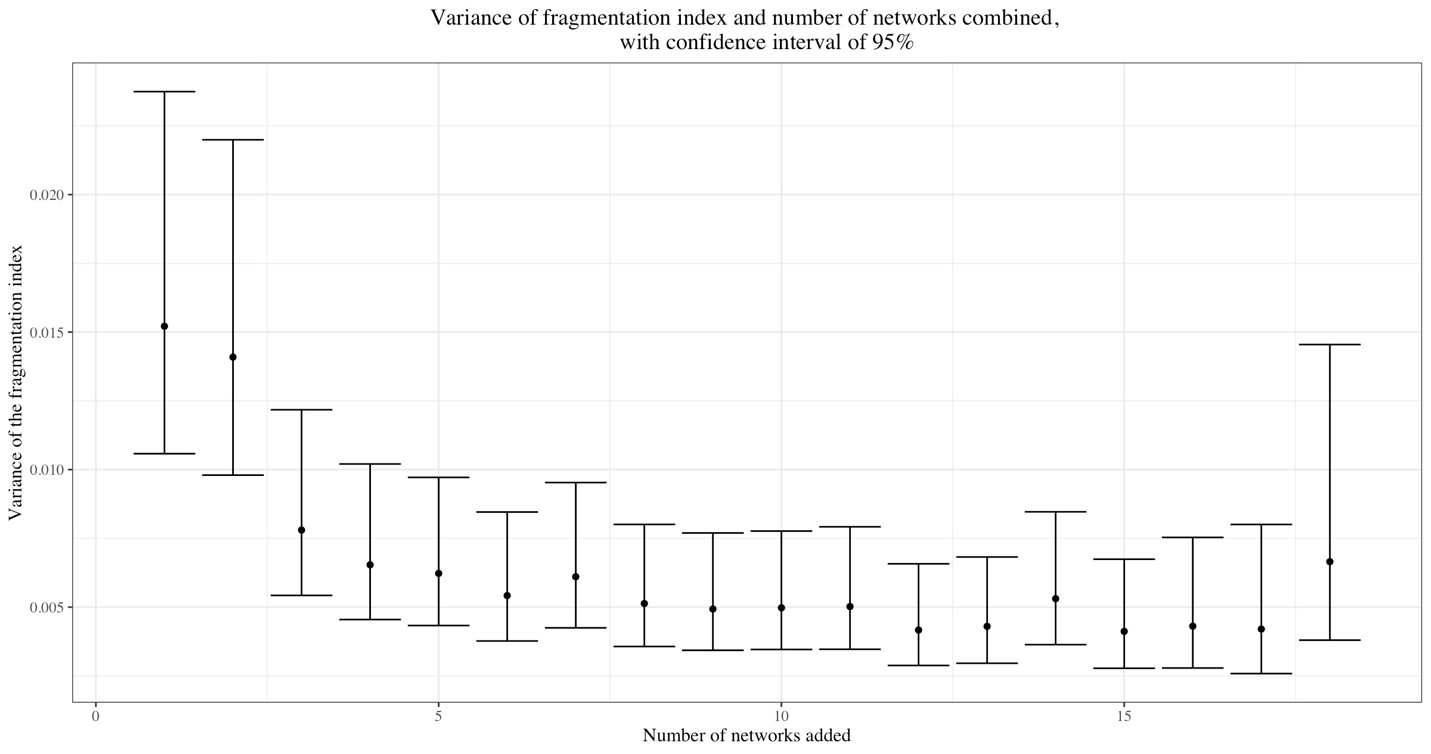
Appendix Plot 5.4. Number of networks added and the variance of fragmentation index.

1. **Number of accounts selected to construct the network and edge coverage**

Finally, we show how the number of selected accounts affects the edge coverage. Firstly, we calculated the edge coverage for each town. Appendix plot 5.5 shows the confidence intervals for edge coverage and the number of networks combined (which is equal to the number of accounts selected minus one). From the plot, we observe that after 14 combined networks, the average edge coverage in the sample is over 15%. However, even if all 18 accounts are chosen there are towns for which the edge coverage is lower than 15% which is shown by the confidence interval.


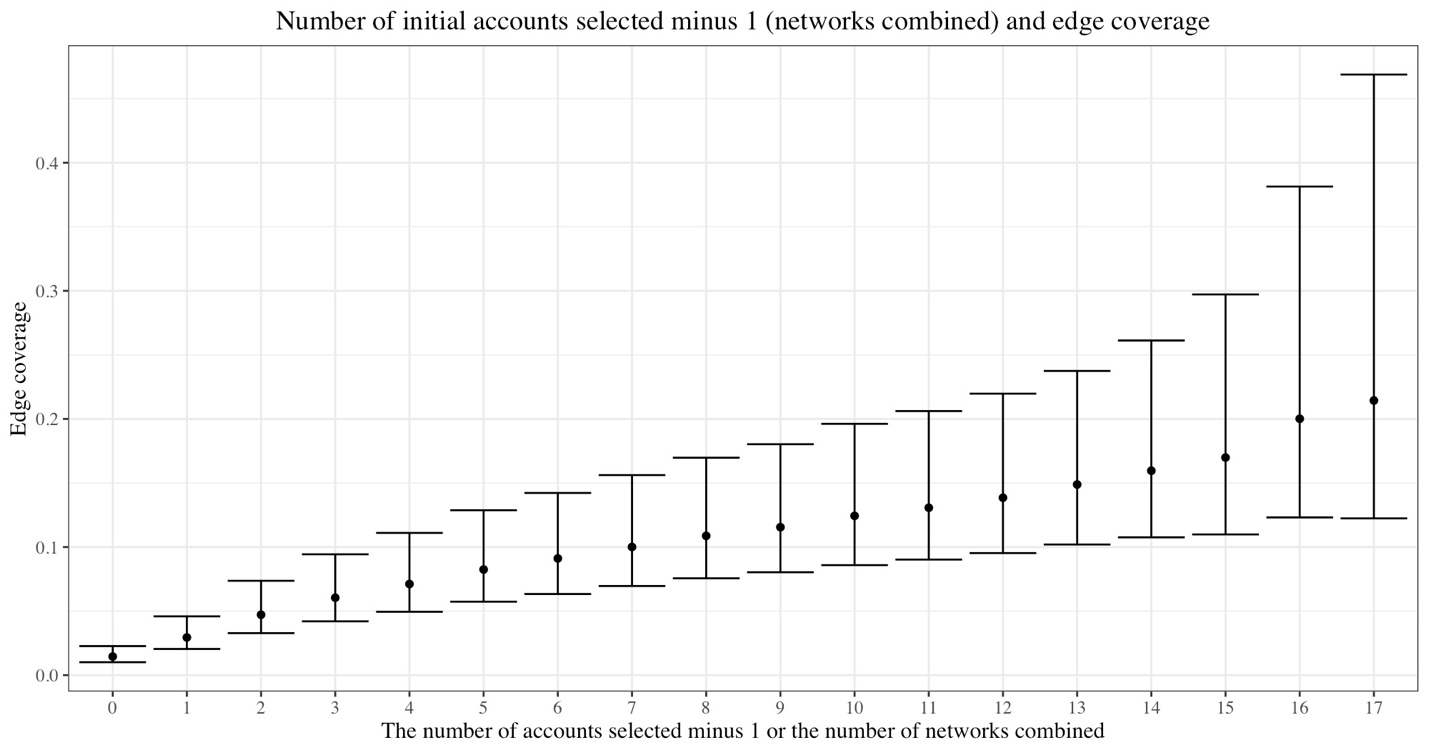
Appendix Plot 5.5. A number of networks added and the edge coverage. 95% confidence intervals.

Appendix 5.6 shows the distribution of towns by edge coverage by the number of networks combined. The plot shows that the share of towns with over 15% edge coverage peaked at 16 16 networks combined at 58%. Moreover, multiple towns are below 15% edge coverage which shows that the 18 accounts selected do not always sample enough edges to eradicate sampling bias.
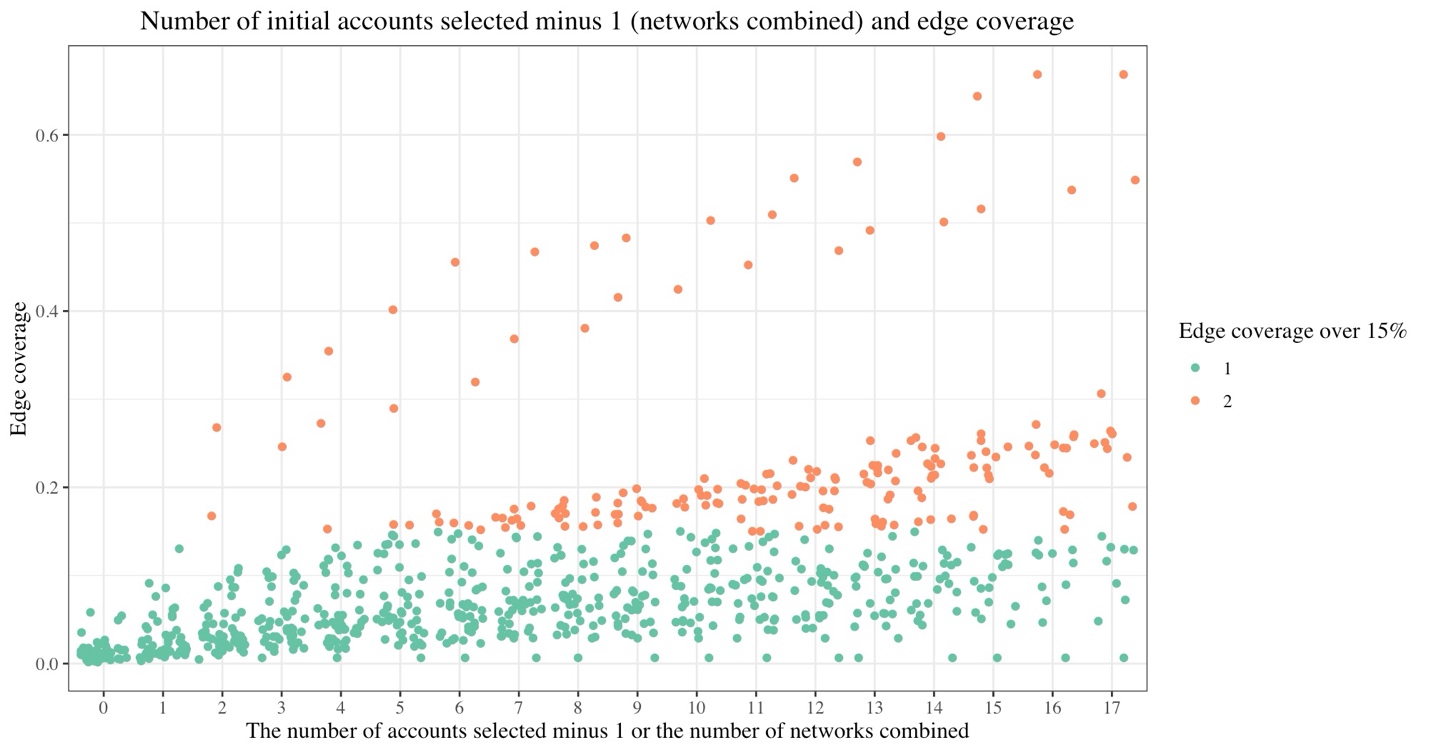


Appendix Plot 5.5. A number of networks added and the edge coverage, color by town with edge coverage over 15%
