# Supplementary material for: Modularity of Online Social Networks and COVID-19 Misinformation Spreading in Russia: Combining Social Network Analysis and National Representative Survey
Source: JMIR Infodemiology. 2025 Jun 26;5:e58302. doi: 10.2196/58302 (PMC12246759; doi:10.2196/58302)
Supplement: Multimedia Appendix 6 [file infodemiology_v5i1e58302_app6.docx]

In this appendix, robustness checks are conducted on the sample of towns where the average edge coverage exceeds 15%. We have excluded towns with the highest risk of measurement bias. This was one by placing towns in ascending order by edge coverage and removing ones with the lowest edge coverage to the point the average edge coverage exceeds 15%. The new dataset included 149 towns out of 166 with the average edge coverage of 15.04%. The sample includes 13074 observations. Consequently, our new dataset is expected to be less susceptible to biases stemming from sampling measurement errors, as an edge coverage of 15% is deemed advisable.

Appendix Table 7.1 shows a linear regression model that looks at the relationship between the fragmentation index and how information spreads. We find that this relationship does not hold for fake statements, but it does hold for true statements.

|  | | |
| --- | --- | --- |
|  | Dependent variable: | |
|  |  | |
|  | Share of encountered fake statements about COVID-19 | Share of encountered true statements about COVID-19 |
|  | (1) | (2) |
| Fragmentation index | -0.001  (0.004)  *P* = .8026 | -0.128  (0.035)  *P* = .0003 |
| Size of the network | -0.026  (0.008)  *P* = .0012 | -0.009  (0.008)  *P* = .2606 |
| Vkontakte use dummy | 0.046  (0.006)  *P* = .0000 | 0.027  (0.005)  P = .0000 |
| Socioeconomic town level controls: | + | + |
| Individual-level controls: | + | + |
| Observations | 13,074 | 13,074 |
| R^2^ | 0.015 | 0.034 |
| Note: | Exact P-values are reported | |
|  | Robust standard errors in brackets by town. Socioeconomic town level controls include: the natural log of wages 2019, and the natural log of population 2019. Individual level controls include: gender dummy, education level, household income, age, fear of COVID-19, a dummy variable for household experience with COVID-19, institutional trust measured as a trust to the president, use of TV | |

Appendix table 7.1 Information spreading. Sample with an average edge coverage of over 15%

In Appendix Table 7.2, we look at how fragmentation affects attitudes towards statements about COVID-19 in a sample where edge coverage exceeds 15%. All significant results from the main study remain the same. Additionally, some previously insignificant relationships have now become significant. For example, we see a positive link between town-level fragmentation and people's average attitudes towards fake statements about COVID-19, as well as the percentage of fake statements that respondents agree with. Our checks show that these results are strong and likely reflect an existing association.

|  | | | | | | |
| --- | --- | --- | --- | --- | --- | --- |
|  | *Dependent variable:* | | | | | |
|  | Average attitude to true statements about COVID-19 | Average attitude to fake statements about COVID-19 | Share of true statements agree | Share of fake statements agree | Difference in attitude between fake and true statements | Misinformation error |
|  | (1) | (2) | (3) | (4) | (5) | (6) |
| Fragmentation index | -0.790  (0.280)  *P* = .0048 | 0.922  (0.380)  *P* = .0153 | -0.134  (0.035)  *P* = .0001 | 0.060  (0.022)  *P* = .0064 | 1.712  (0.494)  *P* = .0005 | 0.057  (0.030)  *P* = .0575 |
| Size of the network | 0.124  (0.059)  *P* = .0356 | 0.101  (0.080)  *P* = .2068 | 0.014  (0.008)  *P* = .0801 | -0.003  (0.005)  *P* = .5485 | -0.023  (0.104)  *P* = .8250 | -0.007  (0.006)  *P* = .2434 |
| Vkontakte use dummy | 0.044  (0.039)  *P* = .2593 | -0.119  (0.051)  *P* = .0196 | 0.020  (0.005)  *P* = .0001 | 0.009  (0.003)  *P* = .0027 | -0.162  (0.067)  *P* = .0156 | 0.018  (0.004)  *P* = .0000 |
| Socioeconomic town level controls: | + | + | + | + | + | + |
| Individual-level controls: | + | + | + | + | + | + |
| Observations | 13,074 | 13,074 | 13,074 | 13,074 | 13,074 | 13,074 |
| R^2^ | 0.073 | 0.054 | 0.056 | 0.024 | 0.066 | 0.027 |
| *Note:* | Exact P-values are reported | | | | | |
|  | Robust standard errors in brackets by town. Socioeconomic town level controls include: the natural log of wages 2019, and the natural log of population 2019. Individual level controls include: gender dummy, education level, household income, age, fear of COVID-19, a dummy variable for household experience with COVID-19, institutional trust measured as a trust to the president, use of TV | | | | | |

Appendix table 7.2 Attitude to statements. Sample with an average edge coverage of over 15%
