# Supplementary material for: Modularity of Online Social Networks and COVID-19 Misinformation Spreading in Russia: Combining Social Network Analysis and National Representative Survey
Source: JMIR Infodemiology. 2025 Jun 26;5:e58302. doi: 10.2196/58302 (PMC12246759; doi:10.2196/58302)
Supplement: Multimedia Appendix 7 [file infodemiology_v5i1e58302_app7.docx]

Demographics are compared between data collected from networks and poll data. This section outlines the data collection procedure.

We used preserved VK IDs of accounts for analysis, focusing on Russian towns as of June 2023. On March 18, 2025, we sampled 1,000 accounts from each town. Most networks had over 1,000 nodes, so for smaller networks, all nodes were included. In total, we sampled 166600 accounts from 168 towns.

Using the VK API, we obtained public information for each sampled ID. While some details are available for non-public accounts, most information remains hidden. On average, 25.7% of sampled accounts were private.

To determine user age, we calculated the years between the birthday and June 18, 2023. Many users only published their birthdates without the year, leading to incomplete age data. We excluded accounts claiming to be over 99 years old; 61.9% of open accounts had their birth year published.

For education data, we noted unique university IDs. Users with no higher education information had an ID of 0. We found that 50.3% of open accounts completed the higher education section.

Appendix Table 6.1 shows demographic averages: 61.4% of survey respondents were women, compared to 59.3% of VK accounts, with average ages of 37.35 and 36.10, respectively. Notably, 59.5% of respondents had completed university, while only 32.3% of VK users reported higher education.

The quality of demographic data from the network is limited, as users aren't required to share their information, leading to potential omissions. In summary, the share of women is slightly higher in our poll, but the proportion of individuals with higher education is significantly greater, suggesting potential underrepresentation of those without higher education in the poll sample.

|  | Share of women | Age | Share with higher education |
| --- | --- | --- | --- |
| Respondents from the poll data | 0.614 | 37.35 | 0.595 |
| VK users, network data | 0.593 | 36.10 | 0.323 |
| Average number of VK users in the sample in each town who published information about certain demographic characteristic | 991.67 | 455.02 | 370.58 |
| T-test for differences in means, p-value | *P* = .0011 | *P* = .0000 | *P* = .0000 |

Appendix table 6.1 Comparing demographics from poll and from the network data.
